# Activation of Small Molecules by Modified Dodecaborate Anions

**DOI:** 10.1021/acs.jpca.3c07361

**Published:** 2024-03-08

**Authors:** Mehmet Emin Kilic, Puru Jena

**Affiliations:** Department of Physics, Virginia Commonwealth University, Richmond, Virginia 23284-2000, United States

## Abstract

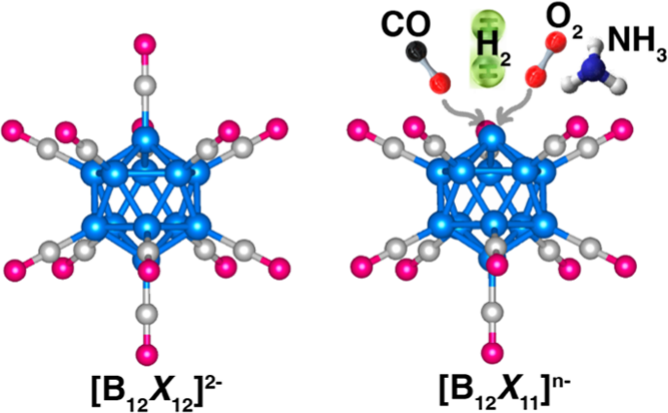

Two of the basic requirements of a good catalyst are that molecules be bound to it with energies intermediate between physisorption and chemisorption and be simultaneously activated in the process. Using density functional theory, we have studied the interaction of small molecules such as H_2_, O_2_, N_2_, CO_2_, CO, and NH_3_ with modified dodecaborate anion [B_12_H_12_]^2–^, namely, [B_12_X_11_]^−^ and [B_12_X_11_]^2–^ (X = H, F, CN). Calculations of the structure, stability, and electronic properties of these species interacting with the above molecules show that they meet the above requirements. In addition, [B_12_X_11_]^2–^ (X = F, CN) species are not only more stable than [B_12_X_11_]^−^ species but also bind to O_2_ more strongly than their monoanion counterparts.

## I Introduction

Dodecaborate ions, namely, [B_12_H_12_]^2–^, were predicted and synthesized more than half a century ago.^1−3^ These are cage clusters composed of 12 B atoms occupying the vertices of an icosahedron with 12 hydrogen atoms radially bonded to the B atoms. Over the past several decades, considerable efforts have been made to study their properties not only by changing the ligands from hydrogen to halogen, pseudo-, and superhalogen moieties but also by replacing the core B atoms with Be, C, and N.^4−13^ The stability of these species was explained by Wade^14^ and later extended by Mingos^15^ and Jemmis.^16^ Commonly known as Wade’s rule, it is based on the polyskeletal electron pair theory (PSEPT) which states that [B*_n_*H*_n_*]^2–^ (*n* ≥ 6) requires (*n* + 1) pairs of electrons for its stability. Despite its small size (radius of 5.81 Å), [B_12_H_12_]^2–^ is stable as a dianion in the gas phase with the second electron bound by 0.9 eV.^17−19^ It was later shown that the binding energy of the second electron, known as the second electron affinity, can be higher if the H atoms are replaced by halogen atoms; the second electron affinity of [B_12_Br_12_]^2–^ reaches as high as 2.71 eV.^10^ Recently, Zhao et al. predicted that if a halogen can be replaced by a superhalogen, CN, the second electron affinity could be even higher, namely, 5.3 eV.^20^ This unprecedented high binding energy of the second electron was later experimentally confirmed to be 5.5 eV, making [B_12_(CN)_12_]^2–^ the most stable dianion to date in nature.^21^ In the same experiment, the authors showed that if one of the CN ligands is detached, the uncoordinated or “naked” B atom (i.e., the B atom without a ligand) in [B_12_(CN)_11_]^−^ can bind noble gas atom Ar at room temperature. Using density functional theory, the authors showed that the “naked” B atom carries a positive charge while the excess electron in [B_12_(CN)_11_]^−^ is distributed mostly over the CN ligands. The electrophilic nature of the “naked” B atom was shown to be responsible for Ar binding. A few years earlier, Rohdenburg et al. had also shown that the electrophilic B atom in [B_12_Cl_l1_]^−^ could bind noble gas atoms, Kr and Xe, spontaneously at room temperature.^22^ Mayer et al. later observed binding of CO with [B_12_X_11_]^−^ (X = F, Cl, Br, I, CN). They showed that the strong electrostatic effect of the “naked” boron overcompensates the weak π-backbonding^23^ and, therefore, a blue shift of the vibrational frequencies was observed.

In a recent article,^24^ we showed that if one of the ligands *X* from [B_12_X_12_]^2–^ (X = F, CN) could be removed in *neutral* form, the resulting cluster, [B_12_X_11_]^2–^, can remain stable against the detachment of the second electron. This is consistent with an earlier experimental observation of [B_12_Br_11_]^2–^.^11^ Here, we show that [B_12_X_11_]^2–^ (X = F, Cl, Br, I, CN) clusters are electronically more stable than their corresponding monoanions [B_12_*X*_11_]^−^. In addition, [B_12_X_11_]^2–^ (X = F, CN) binds to O_2_ more strongly than its monoanionic counterpart.

Note that whether the ligand X in [B_12_X_12_]^2–^ would detach in anionic or neutral form would depend not only on which detachment path costs the least energy but also on the energy barrier the ligand needs to cross in the process. From the energy cost point of view, the ligand would detach in the anionic form if the electron affinity of the ligand is higher than that of the second electron affinity of [B_12_X_11_]^2–^. In Figure S1, we plot the energy cost to remove the ligand X in neutral as well as in the anionic form. Note that the energy cost to remove the ligand in the anionic form is less than that in the neutral form, leaving behind [B_12_X_11_]^−^. However, this is not always the case. As mentioned before, the existence of [B_12_Br_11_]^2–^, observed experimentally,^11^ implies that the energy barrier the ligand needs to cross, commonly known as the repulsive Coulomb barrier, would also play a role.

We had earlier shown that [B_12_X_11_]^2–^ (X = F, CN) can activate CO_2_ and N_2_ more strongly than their monoanionic counterpart, [B_12_X_11_]^−^.^24^ To understand the underlying reason for this enhanced reactivity, we examined the distribution of the second electron in [B_12_X_11_]^2–^. This was found to primarily go to the “naked” B site, reducing its charge from 0.70 e in [B_12_(CN)_11_]^−^ to 0.26 e in [B_12_(CN)_11_]^2–^, making the “naked” B less electrophilic. The reason for [B_12_X_11_]^2–^ to be more reactive than [B_12_X_11_]^−^ is that the former is a radical ion with an unpaired electron and its second electron affinity, namely, 3.17 eV, is smaller than the ionization potential of any atom in the periodic table.

In this paper, we show that [B_12_X_11_]^2–^ (X = F, CN) clusters bind to O_2_ more strongly than [B_12_X_11_]^−^ while reverse is the case for the binding of CO and NH_3_. In what follows, we describe our computational procedure and discuss our results. Our reasons for focusing on the reactions with H_2_, O_2_, CO, and NH_3_ are outlined in Section III.

## II Computational Methods

The geometries and total energies of [B_12_*X*_12_], [B_12_X_11_]^*n*−^, and [B_12_X_11_]^*n*−^ (*n* = 0, 1, 2; X = H, F, CN) clusters interacting with small molecules, Y = H_2_, O_2_, CO, and NH_3_, are calculated using density functional theory (DFT) and the B3LYP exchange-correlation functional.^25^ We used the Gaussian 16 code^26^ with the 6-31+G(d,p) basis set for all atoms as this choice has led to good agreement between theory and experiment.^24^ All the structures are fully optimized without any symmetry constraint. The convergence in the total energy and force are set at 10^–6^ eV and 10^–2^ eV Å^–1^, respectively. To determine the preferred spin multiplicities of [B_12_X_11_]^*n*−^ (*n* = 1, 2) clusters, we calculated the total energies of the species for the two lowest spin multiplicities, i.e., singlet and triplet states for an even number of electrons and doublet and quartet states for those containing an odd number of electrons. With the exception of [B_12_(CN)_11_O_2_]^−^, all clusters with an even (odd) number of electrons are found to be in the spin singlet (doublet) state. Note that all the molecules studied here have singlet spin states except O_2_, which has a spin triplet ground state with the singlet state lying 1.67 eV above the triplet state. Hence, O_2_ is known as a diradical. We will discuss the preferred spin multiplicities of [B_12_X_11_O_2_]^*n*−^ (*n* = 1, 2) in more detail in the following section.

The first and second electron affinities (EA_1_ and EA_2_) of these species are calculated using the following equations:
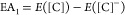
1
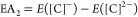
2

The terms *E*([C]), *E*([C]^−^), and *E*([C]^2–^) refer to the total energy of the neutral, monoanionic, and dianionic form of C, respectively, where C represents either [B_12_X_12_] or [B_12_X_11_] with or without adsorbed gas molecules, Y. The energy costs, Δ*E*^0^ and Δ*E*^–^, to detach a ligand in a neutral state (X) or in a monoanionic state (X^–^), respectively, are calculated as
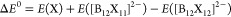
3
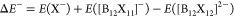
4

## III Results and Discussion

In the following we provide the results of our calculations for [B_12_X_12_] and [B_12_X_11_] with or without the adsorbed gas molecules, Y.

### III.I Geometries and Electronic Structure of [B_12_X_12_]^2–^, [B_12_X_11_]^*n*−^ (*n* = 0, 1, 2) (X = H, F, CN)

#### III.I.I [B_12_X_12_]

**Figure 1:**
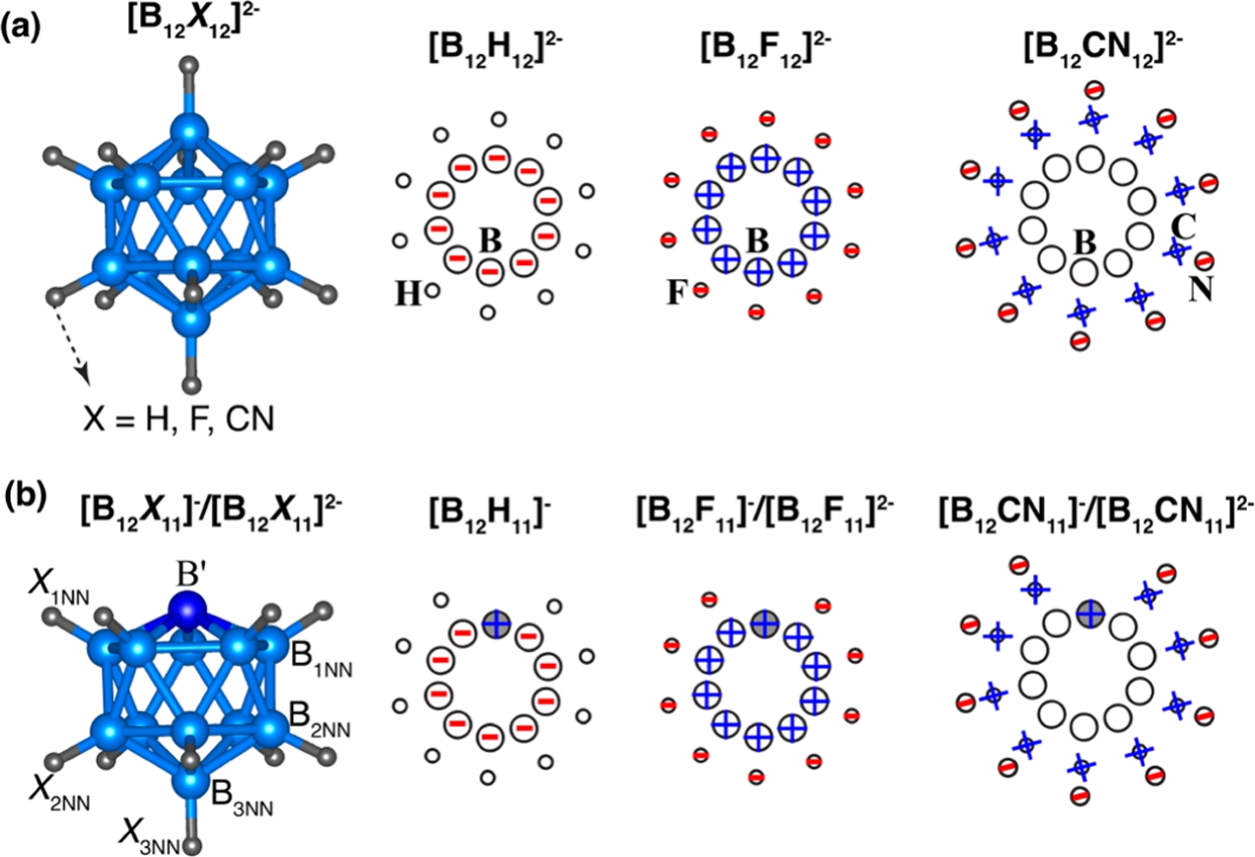
Optimized atomic structures of (a) [B_12_X_12_]^2–^ and (b) [B_12_X_11_]^−^ and [B_12_X_11_]^2–^. B′, B_1NN_, B_2NN_, and B_3NN_ represent the “naked” boron atom (which lost a ligand) and its first, second, and third nearest neighbors, respectively. X_1NN_, X_2NN_, and X_3NN_ represent the ligand atoms bound to B_1NN_, B_2NN_, and B_3NN_, respectively.

As mentioned before, [B_12_X_12_]^2–^ clusters consisting of 12 boron and 12 X (X = H, F, and CN) ligands have been studied extensively and are known to have an icosahedron geometry (Figure 1a). To validate our computational method, we first calculated the ground-state geometries and total energies of [B_12_X_12_]^*n*−^ (*n* = 0, 1, 2) clusters and compared them with previous calculations.^9,13,17,20,21^ The dianionic states of [B_12_X_12_]^2–^ are found to have the lowest energies, and thus the highest stability. We also note that, for each ligand X, the dianion is in the spin singlet state and has lower energy compared to that in the spin triplet state. All these findings adhere to Wade’s electron-counting rule, which states that [B_12_X_12_] clusters are most stable as a dianion.

The calculated first (EA_1_) and second (EA_2_) electron affinities of [B_12_X_12_] are summarized in Table 1 and compared with previous calculations. The EA_1_ values of B_12_X_12_ are 4.77, 5.75, and 8.57 eV for X = H, F, and CN, respectively. The second electron affinities of B_12_X_12_ with the EA_2_ values of 0.86, 1.58, and 5.28 eV for X = H, F, and CN, respectively, not only demonstrate their stability as a dianion but also that ligands have a substantial effect on their stability. These findings are consistent with theoretical results reported earlier, as well as with the experiment results presented in Table 1.^9,13,17,20,21^ For the cyano ligand (CN), the total energy of [B_12_(CN)_12_]^2–^ (where the C atom of CN is bound to the boron atom) is 4.03 eV lower in energy than that of [B_12_(NC)_12_]^2–^ (where the N atom of CN is bound to the boron atom). As the binding of the C atom of the CN bound to B is more favorable than that of the N atom, we only study [B_12_(CN)_12_]^2–^ clusters in the following.

**Table 1:** First (EA_1_) and Second (EA_2_) Electron Affinities in eV of [B_12_X_12_], [B_12_X_11_], and [B_12_X_11_Y] where Y = H_2_, O_2_, CO, and NH_3_^a^

	[B_12_X_12_]	[B_12_X_11_]	[B_12_X_11_Y]			
			Y = H_2_	Y = O_2_	Y = CO	Y = NH_3_
	EA_1_ (EA_2_)	EA_1_ (EA_2_)	EA_1_ (EA_2_)	EA_1_ (EA_2_)	EA_1_ (EA_2_)	EA_1_ (EA_2_)
X = H	4.77, 4.53^b^, 4.55^c^, 4.57^d^ (0.86, 0.81^b^, 0.96^c^, 0.86^d^, 0.9^g^)	5.30 (−1.81)	5.07	5.42	5.24	4.53
X = F	5.75, 5.69^b^,^c^ (1.58, 1.30^b^,^c^)	5.79 (0.83)	5.60 (−0.32)	5.91 (1.96)	5.67 (−0.66)	5.12 (−1.61)
X = CN	8.57, 8.71^b^, 8.56^d^ (5.28, 5.26^b^, 5.28^d^, 5.45^e^, 5.53^f^)	8.49 (3.17)	8.36 (2.09)	8.33 (4.69)	8.33 (1.78)	8.09 (−0.39)

a The EA_2_ values are given in parentheses.

b Ref (9) with PBE0/def2-TZVP.

c Ref (13) with PBE0/def2-TZVP.

d Ref (20) with B3LYP/6-31+G(d,p).

e Ref (9) with B3LYP/6-331++G(2d, 2p).

f Ref (9) with PBE0/def2-TZVPPD.

g Ref (17) with B3LYP/6-311G+(d).

To further investigate the influence of the X ligand on the geometrical parameters, we calculated the B–B and B–X bond lengths in [B_12_X_12_]^2–^ dianions (see Table 2). The B–B bond lengths are 1.787, 1.797, and 1.798 Å for X = H, F, and CN, respectively. This shows that the variation of X has only a marginal effect on the B–B distances. In contrast, the B–X distances vary significantly. The calculated B–X bond lengths are found to be 1.206, 1.396, and 1.543 Å, for X = H, F, and CN, respectively, and are consistent with the sum of the covalent radii of the corresponding elements B and X.

**Table 2:** Structural Parameters (Bond Distances, *d*, in Å) and Natural Charges (*q*) in Electrons of Boron and X Ligands (X = H, F, and CN) in [B_12_X_12_]^2–^, [B_12_X_11_]^−^, and [B_12_X_11_]^2–^a

X	[B_12_X_12_]^2–^	[B_12_X_11_]^−^/[B_12_X_11_]^2–^				
	*d*(B–B)	*d*(B′–B_1NN_)	*d*(B_1NN_–B_1NN_)	*d*(B_1NN_–B_2NN_)	*d*(B_2NN_–B_2NN_)	*d*(B_2NN_–B_3NN_)
–H	1.787	1.696	1.833	1.791	1.801	1.781
–F	1.797	1.736/1.753	1.856/1.800	1.802/1.798	1.815/1.804	1.802/1.796
–CN	1.798	1.715/1.768	1.845/1.793	1.806/1.804	1.815/1.800	1.801/1.798

	*d*(B–X)	*d*(B′)	*d*(B_1NN_–X_1NN_)	*d*(B_2NN_–X_2NN_)	*d*(B_3NN_–X_3NN_)
–H	1.206		1.192/	1.194/	1.194
–F	1.396		1.372/1.400	1.376/1.397	1.377/1.397
–CN	1.543		1.533/1.547	1.536/1.544	1.537/1.544

	*q*(B)	*q*(B′)	*q*(B_1NN_)	*q*(B_2NN_)	*q*(B_3NN_)
–H	–0.19	+0.45/	–0.22/	–0.17/	–0.21/
–F	+0.34	+0.37/–0.08	+0.36/+0.33	+0.36/+0.35	+0.35/+0.34
–CN	–0.03	+0.70/0.26	–0.08/–0.10	–0.02/–0.03	–0.05/–0.04

	*q*(X)	*q*(X_1NN_)	*q*(X_2NN_)	*q*(X_3NN_)
–H	+0.02	+0.07/	+0.06/	+0.07/
–F	–0.51	–0.48/–0.52	–0.48/–0.51	–0.48/–0.51
–CN	–0.14	–0.11/–0.07	–0.11/–0.08	–0.10/–0.08

a B′, B_1NN_, B_2NN_, and B_3NN_ represent boron atoms with the absence of X ligand and first, second, and third nearest neighbor boron atoms to B′, respectively. X_1NN_, X_2NN_, and X_3NN_ denote ligand atoms attached to B_1NN_, B_2NN_, and B_3NN_, respectively.

To gain insights into the electron distribution within [B_12_X_12_]^2–^, namely, where the negative charges reside, we performed a Natural Bond Orbital (NBO) analysis. Schematic partial charges are illustrated in Figure 1. For X = H, the negative charges are entirely localized on the boron atoms. Each hydrogen atom donates a minute amount of charge of approximately 0.02 e to the boron atoms whereas each boron atom gains 0.19 e. On the other hand, in the case of X = F, each boron atom lost 0.34 e and became positively charged. As the electron affinity of the F atom is higher than that of the boron atom, all the negative charges are transferred to the F ligands and each of them gains 0.51 e. These results agree well with previous results where each B lost 0.3 e and each F gained 0.47 e.^10^ Unlike when X = H and F, for X = CN, boron atoms do not participate in any cooperative charge transfer. Each boron atom gains only 0.03 e in [B_12_(CN)_12_]^2–^; the main contributors to charge transfer are the carbon and nitrogen atoms of the CN ligands.

#### III.I.II [B_12_X_11_]

Next, we focus on [B_12_X_11_] clusters, which are formed by removing one X ligand and generating an uncoordinated boron (referred to as the ‘“naked” boron atom and labeled as B′) (Figure 1b). As discussed before, the ligand, when removed, prefers to detach as a negative ion carrying the excess electron with it. Removing a ligand X in its neutral form would lead to [B_12_X_11_]^2–^. In a recent paper, we showed that [B_12_X_11_]^2–^ dianions (X = F, CN) are not only thermodynamically and dynamically stable but also demonstrate exceptional ability in activating CO_2_ and N_2._^24^ We also noted that the second electron occupies the singly occupied molecular orbital (SOMO) and the spin is strongly localized on the “naked” boron atom. In this work, we systematically study the potential of both [B_12_X_11_]^−^ monoanion and [B_12_X_11_]^2–^ dianion clusters to activate a range of other molecules, Y = H_2_, O_2_, CO, and NH_3_. We examine the role of the electrophilicity of the “naked” boron atom in binding these species.

After full geometry optimization without any symmetry constraint, we found that the “naked” boron atom slightly moved toward the center of the dodecahedral cage, resulting in a shorter distance from B′ to its first nearest neighbor boron atoms (B_1NN_) and an elongation of the bonds between B_1NN_ atoms. It is noted that the changes in the atomic positions of the boron atoms in [B_12_X_11_]^2–^ are marginal when compared with those in [B_12_X_11_]^−^. As a result, structural integrity from [B_12_X_12_]^2–^ to [B_12_X_11_]^−^ and [B_12_X_11_]^2–^ is maintained. All structure parameters are summarized in Table 2.

In addition to the thermodynamical stability, we investigated the dynamical stability of [B_12_X_11_]^−^ monoanion and [B_12_X_11_]^2–^ dianion clusters. The absence of imaginary vibrational frequencies (see Table S3 for details) in these structures confirms that they belong to minima in the potential energy surface. To further study their stability when negatively charged, we calculated their electron affinities which measure the energy gain when an additional electron is added. The calculated EA_1_ values are 5.29, 5.79, and 8.49 eV and the EA_2_ values are −1.81, 0.83, and 3.17 eV for X = H, F, and CN ligands, respectively. The positive electron affinities of [B_12_X_11_]^2–^ (X = F, CN) confirm that these are stable.

To further examine the electronic structure of [B_12_X_11_]^−^ and [B_12_X_11_]^2–^, we studied the distribution of negative and positive charges within [B_12_X_11_] clusters by calculating the NBO charges. The results are given in Table 2. In [B_12_H_11_]^−^, the different boron atoms exhibit the following charges: B′: +0.45 e, B_1NN_: −0.22 e, B_2NN_: −0.17 e, and B_3NN_: −0.21 e. This suggests that B′ loses electrons, while the other boron atoms gain electrons. Their natural electronic states are as follows: B′: 2s^0.65^ 2p^1.88^, B_1NN_: 2s^0.66^ 2p^2.53^, B_2NN_: 2s^0.65^ 2p^2.49^, and B_3NN_: 2s^0.64^ 2p^2.54^. Notably, the differences arise primarily from the 2p orbital of B′. It is worth mentioning that the average natural charge on the H ligands is 0.07 electrons per hydrogen atom, with natural electronic states showing 1s^0.93^. This suggests that hydrogen makes a minimal contribution to the charge transfer. Thus, the polarization in [B_12_H_11_]^−^ primarily results from the presence of the positively charged “naked” boron atom and the negatively charged coordinated boron atoms.

In the case of [B_12_F_11_]^−^, the boron atoms possess charges as follows B′: +0.37 e, B_1NN_: +0.36 e, B_2NN_: +0.36 e, and B_3NN_: +0.35 e per boron atom, while each fluorine ligand carries a charge of −0.48 e. Therefore, the positive and negative charges are distributed forming a core–shell model in [B_12_F_11_]^−^ (see Figure 1b, middle panel). As for [B_12_F_11_]^2–^, the boron atoms possess charges as follows B′: −0.08 e, B_1NN_: +0.33 e, B_2NN_: +0.35 e, and B_3NN_: +0.34 e per boron atom, while all fluorine ligands carry a charge of −0.51 e/–0.52 e each. Thus, the extra electron moved to the “naked” boron atom in [B_12_F_11_]^2–^, nearly neutralizing its charge.

For [B_12_(CN)_11_]^−^, the “naked” boron atom has +0.70 e, making it the most electrophilic among [B_12_H_11_]^−^ and [B_12_F_11_]^−^. On the other hand, the remaining boron atoms exhibit the following charges: B_1NN_: −0.08 e, B_2NN_: −0.02 e, and B_3NN_: −0.05 e, indicating that they do not participate in charge transfer in [B_12_(CN)_11_]^−^, contrary to their behavior in [B_12_H_11_]^−^ and [B_12_F_11_]^−^. Furthermore, the carbon atoms have electronic states of 2s^0.92^ 2p^2.92^ and the nitrogen atoms have electronic states of 2s^1.59^ 2p^3.63^. Due to the higher electronegativity of nitrogen compared to carbon, the shared electrons in the CN ligands are pulled toward the nitrogen atom, resulting in a partial negative charge on the nitrogen atoms (−0.24 e) and a partial positive charge on the carbon atoms (0.13 e) in each CN ligand. Consequently, the negative charges are primarily localized on the nitrogen atoms, with only a small number of electrons being present on the coordinated boron atoms, while the positive charges reside on the carbon atoms. In the case of [B_12_(CN)_11_]^2–^, the boron atoms possess charges as follows B′: 0.26 e, B_1NN_: −0.10 e, B_2NN_: −0.03 e, and B_3NN_: −0.04 e per boron atom, while all CN ligands carry a charge of −0.07 e/–0.08 e each.

### III.II Geometries and Electronic Structure of [B_12_X_11_]^−^ and [B_12_X_11_]^2–^ (X = H, F, CN) Interacting with Small Molecules Y (Y = H_2_, O_2_, CO, NH_3_)

In a recent report, we have shown that [B_12_X_11_]^2–^ (X = F, CN) clusters are able to activate CO_2_ and N_2_ more strongly than [B_12_X_11_]^−^.^24^ Here, we extend these studies to H_2_, O_2_, CO, and NH_3_. From the DFT total energy calculations, we determine the most stable atomic configurations of [B_12_X_11_Y]^−^ and [B_12_X_11_Y]^2–^ where Y = H_2_, O_2_, CO, and NH_3_. These molecules were initially placed in various orientations on the corresponding clusters and the geometries were optimized without any symmetry constraint. In the following, we focus only on the lowest energy configurations. Their *xyz* atomic coordinates, total energies, and vibrational frequencies are presented in Tables S1–S3. To gain insight into the adsorption strengths, we calculated the binding energies, EB_1_ and EB_2_, of the molecules using the following equations:
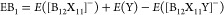
5
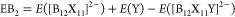
6

Furthermore, we performed vibrational frequency calculations for the lowest energetic configurations and confirmed that all the presented structures are dynamically stable with no negative frequencies.

#### III.II.I Y = H_2_

**Figure 2:**
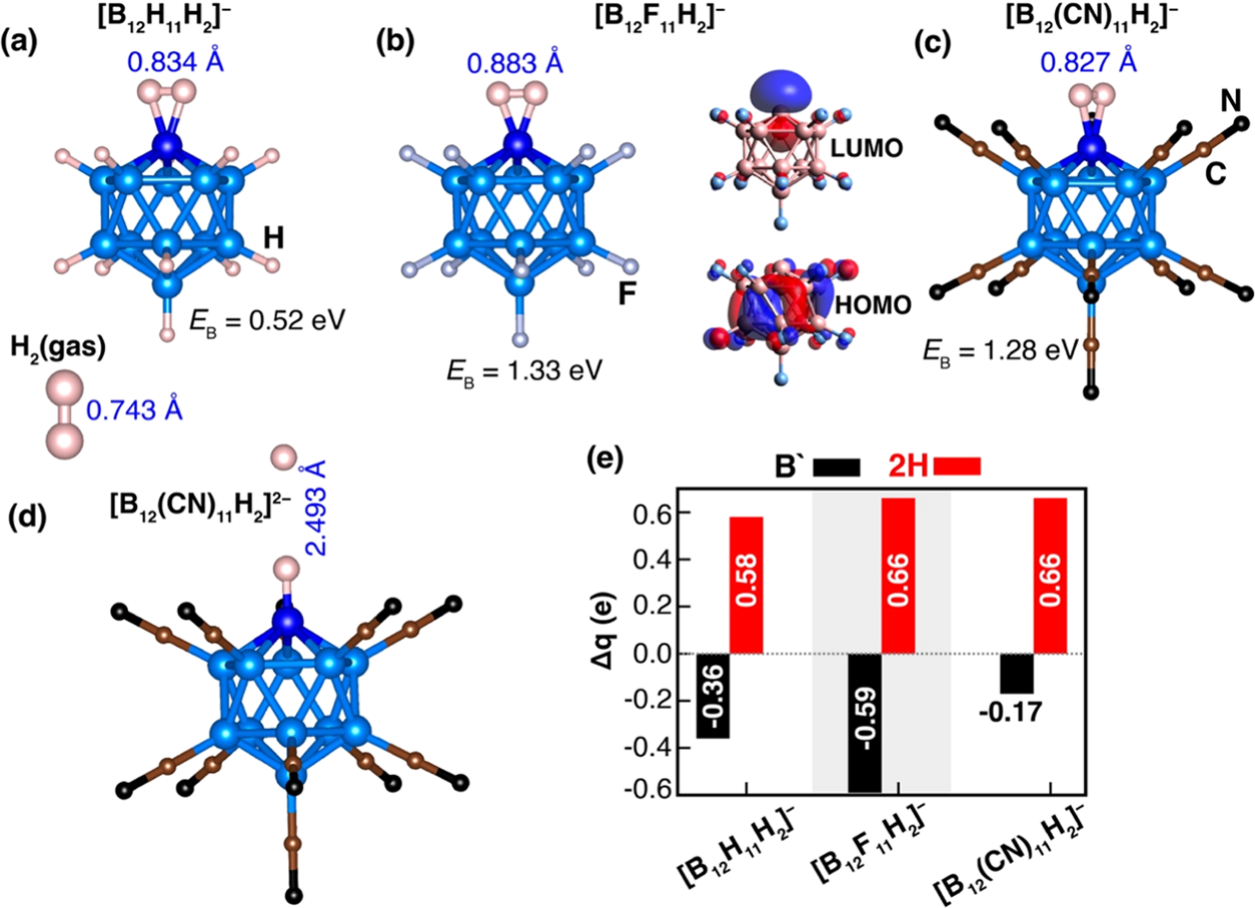
Optimized atomic structures corresponding to the lowest energy configurations for (a) [B_12_H_11_H_2_]^−^, (b) [B_12_F_11_H_2_]^−^, (c) [B_12_(CN)_11_H_2_]^−^, and (d) [B_12_(CN)_11_H_2_]^2–^. Additionally, HOMO–LUMO molecular orbitals for [B_12_F_11_H_2_]^−^ are given in (b). The figure also includes binding energy with D3 dispersions and H–H distance for each corresponding structure. (e) Natural charges on the “naked” boron and hydrogen molecule are depicted by black and red pillars, respectively.

Our initial focus lies in examining whether hydrogen molecules can establish physical or chemical bonds with these clusters. To accomplish this, we investigated the lowest energy geometry of H_2_ when interacting with [B_12_X_11_]^−^ and [B_12_X_11_]^2–^ and calculated the corresponding binding energies. Our DFT calculations reveal that the “naked” boron atom, irrespective of the ligands (H, F, and CN), is the most reactive site for H_2_, resulting in the formation of bonds with two hydrogen atoms. This configuration is referred to as “parallel adsorption”. The lowest energy geometries for [B_12_X_11_H_2_]^−^ are presented in Figure 2. The calculated H_2_ binding energies in [B_12_X_11_H_2_]^−^ are 0.52, 1.33, and 1.28 eV for X = H, F, and CN, respectively. The corresponding H–H distances of 0.834, 0.883, and 0.827 Å are slightly larger than the H–H bond length in the H_2_ molecule, namely, 0.743 Å. These results are consistent with earlier calculations^27^ where the authors computed the H–H bond lengths (binding energies) using the PBE0 functional as 0.850 Å (0.38 eV), 0.910 Å (1.14 eV), and 0.842 Å (1.12 eV) for X = H, F, and CN, respectively.

In the case of the [B_12_F_11_]^2–^ cluster, H_2_ is not bound and when placed on the “naked” B′ atom in [B_12_(CN)_11_]^2–^; it dissociates (see Figure 2d). Thus, [B_12_X_11_]^−^ monoanions are more favorable for H_2_ adsorption when compared with their dianions. The average B′–H_H2_ distances in [B_12_X_11_H_2_]^−^ are calculated to be 1.346, 1.295, and 1.357 Å for X = H, F, and CN, respectively. These distances are larger than the B–H distance in [B_12_H_12_]^2–^ (1.206 Å). Notably, the H_2_ molecule on the [B_12_F_11_]^−^ anion cluster exhibits the closest distance, which correlates with the highest binding energy. As a result, these closely interacting distances may provide a favorable condition for the facile activation of the H_2_ molecule. All these results suggest that the introduction of the “naked” boron strengthens the adsorption toward the H_2_, weakens the H–H bond, and thus enhances the reactivity of the H_2_ molecule.

To further elucidate the nature of interaction and charge transfer in [B_12_X_11_H_2_]^−^, we carried out the NBO charge analysis. The variation of partial charges for the “naked” boron and the adsorbed molecules is presented in Figure 2e. The adsorbed H_2_ molecule is found to have lost 0.58 (0.29 electrons from each hydrogen), 0.66, and 0.66 electrons for X = H, F, and CN, respectively, which are gained by the [B_12_X_11_]^−^ clusters. The natural charges of the “naked” boron in [B_12_X_11_H_2_]^−^ are −0.36, −0.59, and −0.17 e for X = H, F, and CN, respectively. To be more specific, upon H_2_ adsorption, the natural electronic configuration of the “naked” boron atom is changed from 2s^0.65^ 2p^1.88^ to 2s^0.63^ 2p^2.70^ for X = H, from 2s^0.75^ 2p^1.85^ to 2s^0.61^ 2p^2.17^ for X = F, and from 2s^0.60^ 2p^1.67^ to 2s^0.51^ 2p^2.06^ for X = CN. Thus, there is a charge transfer from H_2_ to B′.

To determine and analyze the enhancement in the chemical activity of the H_2_, we examined the molecular orbitals (MOs) of the studied structures before and after they bind to [B_12_X_11_]^−^. The energy gaps between the highest occupied molecular orbitals (HOMO) and lowest unoccupied molecular orbitals (LUMO) for [B_12_X_11_H_2_]^−^ are 5.84, 5.15, and 5.77 eV for X = H, F, and CN, respectively. HOMO/LUMO molecular orbitals for [B_12_F_11_H_2_]^−^ are presented in Figure 2b (right panel), while for the remaining cases, they can be found in Figure S2b. Notably, the HOMO exhibits greater delocalization on the coordinated boron atoms (B_1NN_, B_2NN_, and B_3NN_), whereas the LUMO predominantly resides on both the “naked” boron atom and the H_2_ molecule. This observation underscores the observed charge transfer between the H_2_ molecule and the [B_12_X_11_]^−^ monoanions.

#### III.II.II Y = O_2_

An oxygen molecule (O_2_) is essential in many catalytic oxidation reactions as catalysts can facilitate the transfer of oxygen atoms or molecules to other compounds, leading to their oxidation. For instance, O_2_ reacts with hydrocarbons to produce CO_2_ and H_2_O in the automotive catalytic converter. When O_2_ is activated on a catalyst, it can undergo various chemical reactions depending on the catalyst and the reaction condition. Activating an O_2_ molecule typically means breaking the O–O bond and creating reactive oxygen species that can participate in chemical reactions.^28^ Warneke et al. studied the reaction of O_2_ molecules with *closo*-borate radical anions and showed the formation of very strong B–O bonds.^4^ In experiment, the halogenated *closo*-dodecaborates exhibit exceptional stability against oxidation.^29^

**Figure 3:**
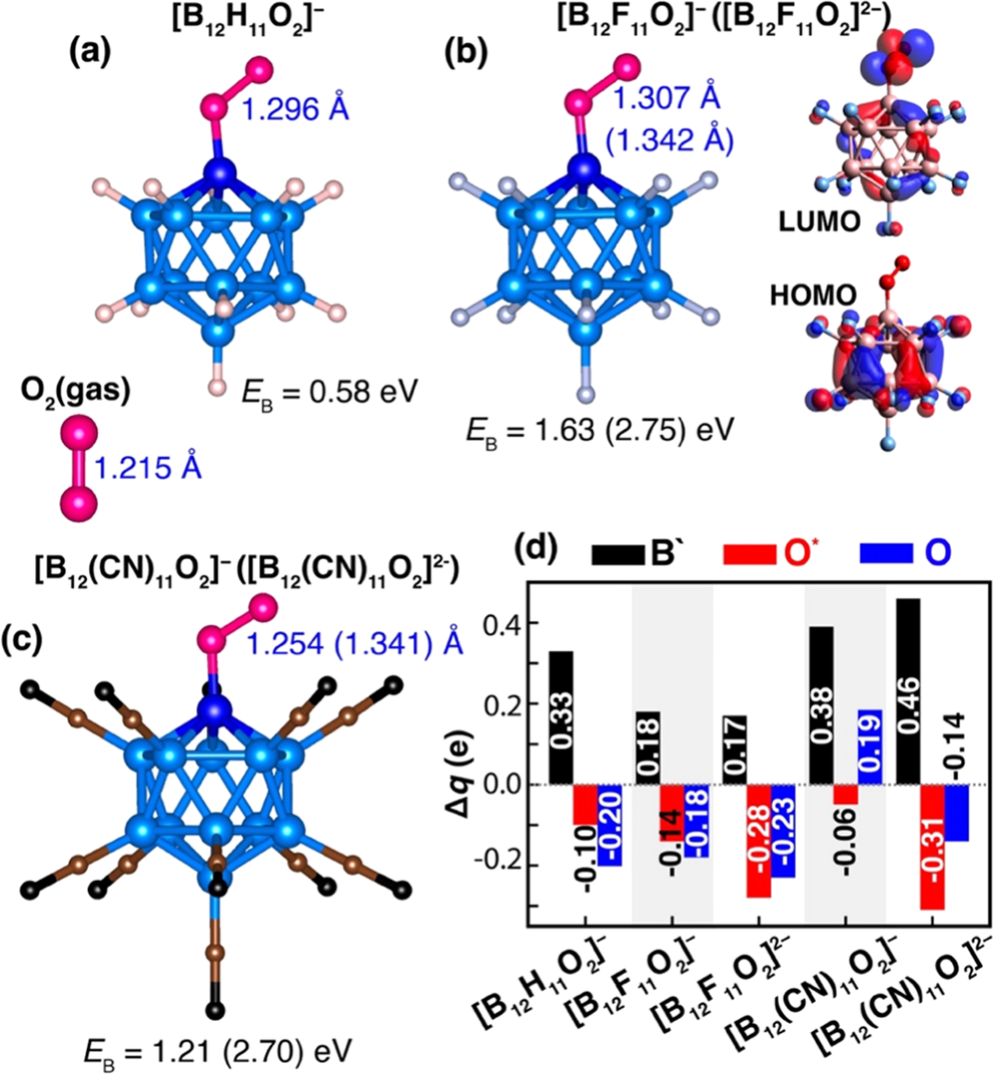
Optimized atomic structures corresponding to the lowest energy configurations for (a) [B_12_H_11_O_2_]^−^, (b) [B_12_F_11_O_2_]^−^ ([B_12_F_11_O_2_]^2–^), and (c) [B_12_(CN)_11_O_2_]^−^ ([B_12_(CN)_11_O_2_]^2–^). NBO charge analysis is presented in (d) where the natural charges of “naked” boron (B′), oxygen (O*) bonded to “naked” boron, and the other oxygen (O) are illustrated by black, red, and blue bars, respectively. Calculated binding energies with D3 dispersions and O*–O distances for each corresponding structure are depicted. The values for the dianions can be found enclosed in parentheses. Additionally, the HOMO–LUMO molecular orbitals for [B_12_F_11_O_2_]^−^ are depicted in (b) (right panel).

We carried out DFT calculations to first examine the activation of O_2_ molecules on [B_12_X_11_]^−^ and [B_12_X_11_]^2–^ clusters. From the energetic screening, the “naked” boron atom, once again, is the most active site for O_2_ binding. The lowest energy configurations of [B_12_X_11_O_2_]^−^ for X = H, F, CN and [B_12_X_11_O_2_]^2–^ for X = F and CN are presented in Figure 3. It can be seen that one oxygen atom of the O_2_ is energetically favored to bind to the “naked” boron atom. Thus, in contrast to the H_2_ binding, O_2_ with the parallel adsorption on both [B_12_X_11_]^−^ and [B_12_X_11_]^2–^ is not favorable. The B′–O* (O* refers to the oxygen atom bonded to B′) bond distances are 1.406, 1.393, and 1.495 Å for X = H, F, and CN in [B_12_*X*_11_]^−^, while they are 1.465 and 1.443 Å for X = F and CN in [B_12_X_11_]^2–^, respectively. Therefore, the adsorbed O_2_ molecule is in close proximity to the [B_12_X_11_]^−^ monoanion as well as to the [B_12_X_11_]^2–^ dianion.

The O_2_ binding energies on the [B_12_X_11_]^−^ monoanion are calculated to be 0.58, 1.63, and 1.21 eV for X = H, F, and CN ligands, respectively, while those on the [B_12_X_11_]^2–^ dianion are 2.75 and 2.70 eV for X = F and CN ligands, respectively. It is interesting to note that the binding energies of O_2_ to [B_12_X_11_]^2–^ are significantly larger than that to [B_12_X_11_]^−^. This is because [B_12_X_11_]^2–^ is a radical ion. These results are consistent with experimental observation^4^ that [B_12_I_11_]^2–^ spontaneously adds an O_2_ molecule. In addition, the preferred spin multiplicities of [B_12_X_11_O_2_]^−^ are singlets for X = H and F with the triplet states lying 0.10 and 0.14 eV above the singlet states, respectively. On the other hand, the preferred spin multiplicity of [B_12_(CN)_11_O_2_]^−^ is a triplet with the singlet state lying 0.29 eV above the triplet state. However, the preferred spin multiplicities of [B_12_X_11_O_2_]^2–^ are doublets for both X = F and CN with the quartet states lying more than 2 eV.

Because of the close distances and strong binding energies between O_2_ and [B_12_X_11_]^−^ ([B_12_X_11_]^2–^), the electrophilic B′ can easily promote the breaking of the strong double bond in O_2_. The O–O bond lengths are calculated to be 1.296, 1.307, and 1.254 Å in [B_12_X_11_]^−^ where X = H, F, and CN, respectively, and 1.342 and 1.341 Å in [B_12_X_11_]^2–^ where X = F, and CN, respectively. These O–O distances are larger than that in the free O_2_ molecule, namely, 1.215 Å. The relatively short B–O* distances and the significantly large O–O* distances suggest that one of the oxygen atoms can be easily detached from the other, thereby allowing it to engage in oxidation reaction. Notably, the greater elongation observed in the O–O bond in the [B_12_X_11_]^2–^ dianion makes it particularly more effective than [B_12_X_11_]^−^ for activating the O_2_ molecule.

Due to the significant electronegativity difference between boron (2.04) and oxygen (3.44), charge transfer is likely to occur from the cluster to O_2_. To determine the charge transfer in [B_12_X_11_O_2_]^−^ and [B_12_X_11_O_2_]^2–^, we calculated the NBO charges; the results are illustrated in Figure 3d. The [B_12_H_11_]^−^ and [B_12_F_11_]^−^ clusters donated 0.30 and 0.32 electrons in total to the O_2_ molecule, respectively, where the O* and O gained −0.10 and −0.20 e in [B_12_H_11_O_2_]^−^ and −0.14 and −0.18 e in [B_12_F_11_O_2_]^−^. However, [B_12_(CN)_11_]^−^ gained 0.13 e in total from the O_2_ molecule where the O* gained −0.055 e and O lost 0.19 e and became positively charged.

In order to assess and analyze the enhanced chemical activity of O_2_, we analyzed the MOs. The calculated HOMO–LUMO gaps in [B_12_X_11_O_2_]^−^ are 2.28, 1.79, and 1.25 eV for X = H, F, and CN, respectively, which are significantly reduced compared to the corresponding values observed in [B_12_X_11_H_2_]^−^. Therefore, the binding of O_2_ to [B_12_X_11_]^−^ clusters leads to a reduction in the gap, enhancing the charge transfer between them. This observation is further supported by an analysis of electron distributions within the HOMO/LUMO orbitals (see Figure 3b right panel and Figure S2c).

#### III.II.III Y = CO

We now shift our focus onto a heteroatomic molecule, carbon monoxide (CO). The importance of CO in some catalytic reactions is that it can act as a poison or inhibitor. It can bind strongly to certain catalyst surfaces and block active sites, thus reducing the catalyst’s ability to function effectively. On the other hand, in certain catalytic processes, CO can be intermediate species that plays a vital role in the overall reaction mechanism. The activation of CO typically involves breaking the strong bond between C and O atoms and creating reactive intermediates that can participate in subsequent reactions. It is worth noting that CO has an exceptional sensitivity to both the binding site’s characteristics and the electron density, which is evident in the C–O stretching frequency.^30^ We recall that Rohdenburg et al. studied the interaction between [B_12_Cl_l1_]^−^ and CO experimentally and observed a positive partial charge at the “naked” boron site through the pronounced blue shift observed in the CO stretching frequency of [B_12_Cl_11_CO]^−^ when compared to free CO, which is strongly influenced by electrostatic effects.^22^ Mayer et al. further observed the strong binding of CO with [B_12_X_11_]^−^ (X = F, Cl, Br, I, CN) and found that the strong electrostatic effect of the “naked” boron overcompensates the weak π-backbonding and therefore a blue shift of the vibrational frequencies was observed.^23^

**Figure 4:**
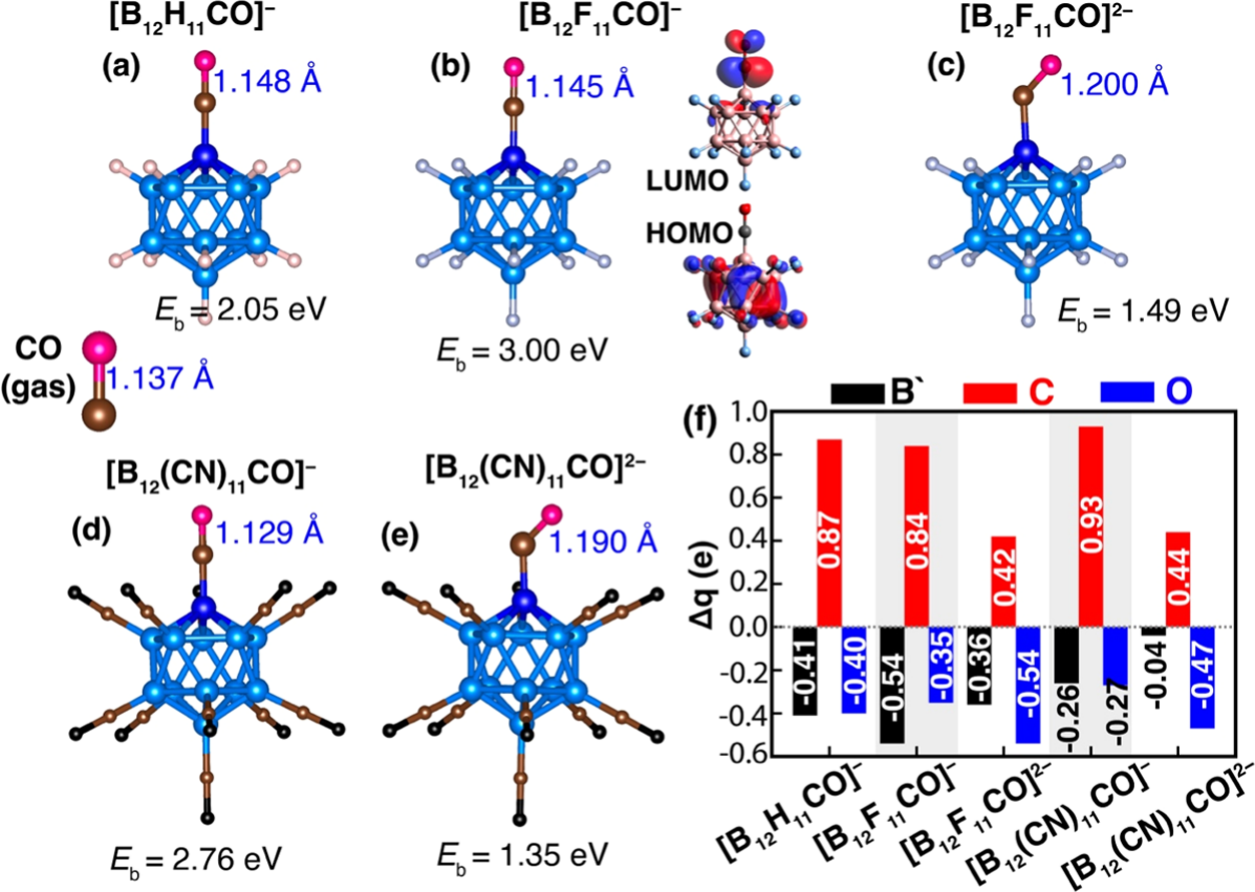
Optimized atomic structures corresponding to the lowest energy configurations of (a) [B_12_H_11_CO]^−^, (b) [B_12_F_11_CO]^−^, (c) [B_12_F_11_CO]^2–^, and (d) [B_12_(CN)_11_CO]^−^, and (e) [B_12_(CN)_11_ CO]^2–^; (f) NBO charge analysis. The natural charges on “naked” boron, carbon, and oxygen are illustrated by black, red, and blue bars, respectively. Calculated binding energies with D3 dispersions and C–O distances for each corresponding structure are depicted. The values for the dianions can be found enclosed in parentheses. Additionally, the HOMO–LUMO molecular orbitals for [B_12_F_11_CO]^−^ are depicted in (b) (right panel).

With this motivation, we performed the DFT calculations to study the activation of CO molecules by both [B_12_X_11_]^−^ and [B_12_X_11_]^2–^ clusters. The carbon atom in the CO molecule is found to bind to the “naked” boron atom in both [B_12_X_11_]^−^ and [B_12_X_11_]^2–^. The most stable structures for [B_12_X_11_CO]^−^ and [B_12_X_11_CO]^2–^ are shown in Figure 4. Note that the CO molecule forms a linear bond with the “naked” boron atom in [B_12_X_11_]^−^, whereas it adopts a tilted configuration when binding to [B_12_X_11_]^2–^. The binding energies of CO in [B_12_X_11_]^−^ are 2.05, 3.00, and 2.76 eV for X = H, F, and CN ligands, respectively. These energies, including the trend, are consistent with prior research^23^ where the authors, using the def2-QZVPP basis set, found [B_12_F_11_]^−^ to exhibit higher binding affinity to CO molecules (2.77 eV) than [B_12_(CN)_11_]^−^ (2.61 eV). In the case of [B_12_X_11_]^2–^, the binding energies are 1.49 and 1.35 eV for X = F and CN, respectively. Because of the stronger binding, [B_12_X_11_] clusters can be considered as promoters and can be added to the list of catalysts to enhance their selectivity and resistance to CO poisoning. That is, we suggest that catalysts can be regenerated by removing the adsorbed CO through [B_12_X_11_] clusters.

To further evaluate the capability of [B_12_X_11_]^−^ and [B_12_X_11_]^2–^ in activating the CO molecule, we calculated the C–O bond lengths. In the case of [B_12_X_11_CO]^−^, we found C–O bond lengths of 1.148, 1.145, and 1.129 Å for X = H, F, and CN ligands, respectively. For [B_12_X_11_CO]^2–^, the C–O bond lengths are found to be 1.200 Å for X = F and 1.190 Å for X = CN ligands. These values exceed the C–O bond length in gaseous CO (1.137 Å), confirming that the CO molecule is activated while interacting with [B_12_X_11_]^−^ and [B_12_X_11_]^2–^ clusters.

The NBO charge analysis in the [B_12_X_11_]^−^ shows that the B′ atom carries a partial negative charge, with values of −0.41, −0.54, and −0.26 electrons for X = H, F, and CN ligands, respectively. Additionally, the carbon atom in CO carries a natural charge of +0.87, +0.84, and +0.93 electrons, while the oxygen atom carries a charge of −0.40, −0.35, and −0.27 electrons for X = H, F, and CN ligands, respectively. As for [B_12_X_11_]^2–^, the “naked” boron B′ possesses −0.36 and −0.04 electrons for X = F and CN ligands, respectively. For X = F, the natural charges of carbon and oxygen in the CO molecule are C: +0.42 e and O: −0.54 e, while for X = CN ligand, these charges are C: +0.44 e and O: −0.47 e.

The HOMO–LUMO gaps are 4.83, 3.92, and 4.26 eV for X = H, F, and CN ligands, respectively. The HOMO–LUMO gap of [B_12_F_11_]^−^ is presented in Figure 4b. Similar to H_2_ and O_2_, the HOMO demonstrates heightened electron delocalization over the coordinated boron atoms, whereas the LUMO predominantly localizes on both the “naked” boron atoms and the CO molecule. The results of the MOs in [B_12_X_11_]^−^ X = H, F, and CN are illustrated in Figure S2d.

#### III.II.IV Y = NH_3_

**Figure 5:**
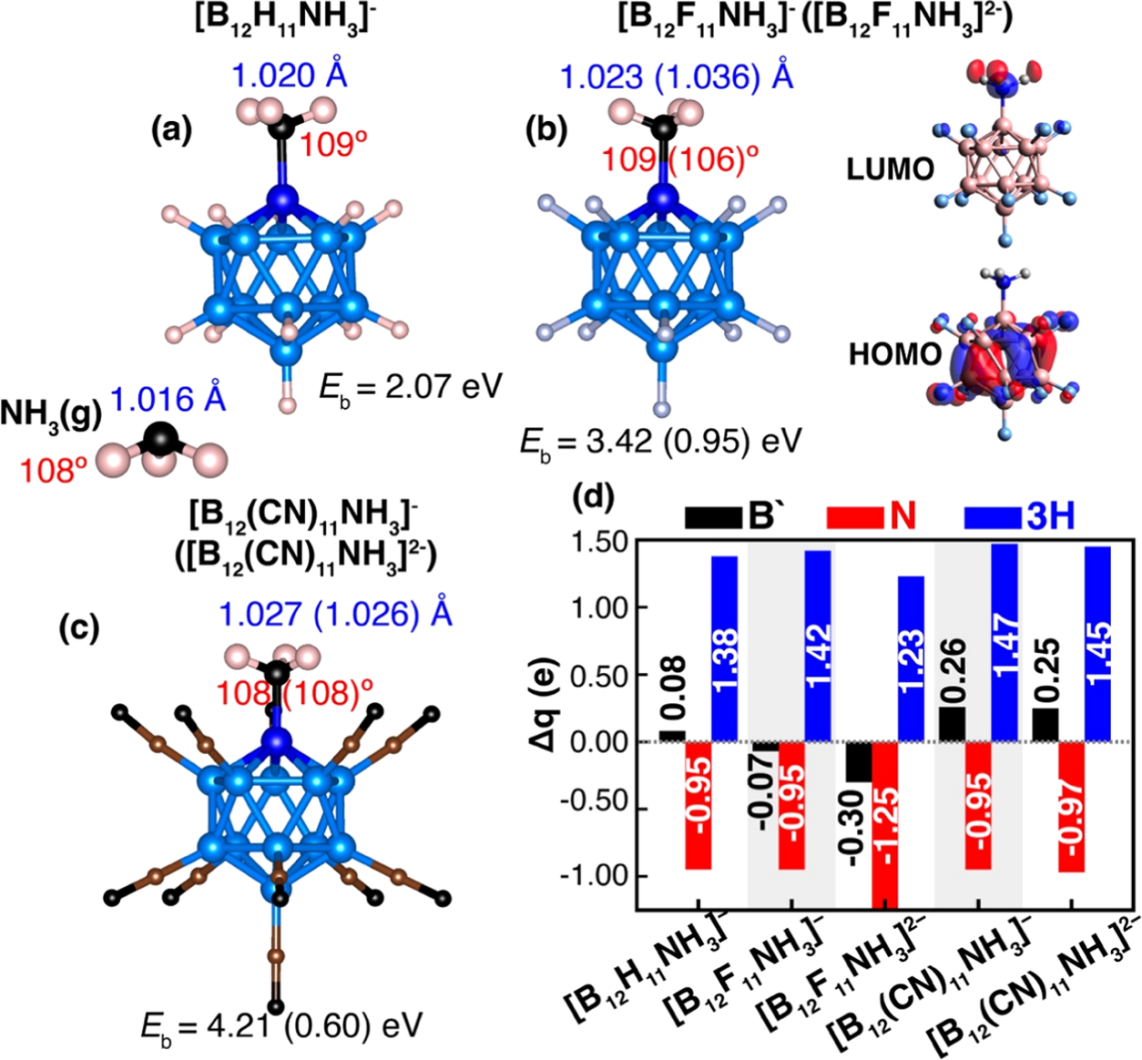
Optimized atomic structures corresponding to the lowest energy configurations of (a) [B_12_H_11_NH_3_]^−^, (b) [B_12_F_11_NH_3_]^−^ ([B_12_F_11_NH_3_]^2–^), and (c) [B_12_(CN)_11_NH_3_]^−^ ([B_12_(CN)_11_NH_3_]^2–^); (d) NBO charge analysis where the partial charges of “naked” boron, nitrogen, and three hydrogen atoms are depicted by black, red, and blue bars, respectively. The calculated binding energies with D3 dispersions and N–H bond distances for each corresponding structure are illustrated. The values for the dianions can be found enclosed in parentheses. Additionally, the HOMO–LUMO molecular orbitals for [B_12_F_11_NH_3_]^−^ are depicted in (b) (right panel).

The process of activating ammonia (NH_3_) involves enhancing its chemical reactivity, typically achieved by either breaking its N–H bonds or forming new chemical bonds. Activated ammonia is often used as a reactant in various chemical reactions and industrial processes. The interaction of NH_3_ molecules with boron clusters has been a subject of study for a long time. For instance, an ammonioborane monoanion, denoted as [B_12_H_11_NH_3_]^−^, was initially synthesized in 1964,^31^ and subsequently, its halogen derivatives have been synthesized.^29,32−34^ In our study, we performed the DFT calculation to investigate the interactions between [B_12_X_11_] clusters and NH_3_. Initially, NH_3_ was placed on different sites on the cluster, but the most stable site is again at the top of B′ with B′ bound to the N atom (see Figure 5). Notably, we predict a strong chemisorption between NH_3_ and [B_12_X_11_]^−^ with the binding energies of 2.07, 3.42, and 4.21 eV for X = H, F, and CN, respectively. These values are higher than the adsorption of NH_3_ on a Pt surface (0.6–1.2 eV).^35^ As for [B_12_X_11_]^2–^, the binding energy is found to be 0.95 and 0.60 eV for X = F and CN ligands, respectively. The distances between B′ and the N atom in NH_3_ are 1.596, 1.564, and 1.563 Å in [B_12_X_11_]^−^ for X = H, F, and CN, respectively, while they are 1.570 and 1.559 Å in [B_12_X_11_]^2–^ for X = F and CN, respectively.

In order to analyze the impact of the strong chemical interactions on the geometry of NH_3_, we calculated the bond lengths and angles within the NH_3_ molecule. The calculated N–H bond length of 1.016 Å, H–N–H bond angle of 108°, and H–N–H–H dihedral angle of 117° in a free NH_3_ molecule agree well with previous results in the literature. Upon NH_3_ adsorption onto [B_12_X_11_]^−^ monoanions, we observed elongation in the N–H bond lengths to 1.020, 1.023, and 1.027 Å, with the corresponding H–N–H bond angles of 109, 109, and 108°, and H–N–H–H dihedral angles of 118, 118, and 116° for X = H, F, and CN, respectively. In the case of [B_12_X_11_]^2–^ dianions, we also observed stretched N–H bond lengths of 1.036 and 1.027 Å for X = F and CN, respectively. The H–N–H bond angles remained at 109, 109, and 108° while the H–N–H–H dihedral angles were 106 and 108°, for X = F and CN, respectively. Consequently, the N–H bond distances in the NH_3_ exhibited small increases when bound to [B_12_X_11_]^−^ monoanions and [B_12_X_11_]^2–^ dianions while the changes in the H–N–H bond angles and H–N–H–H dihedral angles were minimal.

As NH_3_ is a polar molecule with a lone pair of electrons on the nitrogen atom, it is expected that the highly electron-deficient boron (B′) in [B_12_X_11_]^−^ would likely take electrons from NH_3_. In order to investigate the charge transfer taking place between NH_3_ and [B_12_X_11_]^−^ monoanions and [B_12_X_11_]^2–^ dianions, we performed NBO charge analysis (Figure 5d). The results show that the natural charges of B′ in [B_12_X_11_NH_3_]^−^ are +0.08, −0.07, and 0.26 electrons for X = H, F, CN, respectively. Significantly, the nitrogen atom acquired −0.95 electrons while the hydrogen atoms lost +0.46, +0.47, and +0.49 electrons per hydrogen in [B_12_X_11_NH_3_]^−^ for X = H, F, CN, respectively. Concerning [B_12_X_11_NH_3_]^2–^, the charges on B′ are −0.30 and +0.25 electrons, those of nitrogen are −1.25 and −0.97 electrons, and those for hydrogen atoms are +0.41 and +0.49 electrons per hydrogen for X = F and CN, respectively.

The HOMO–LUMO energy gaps are 4.52, 4.29, and 6.30 eV for X = H, F, and CN ligands, respectively. In Figure 5b, the HOMO–LUMO distribution for [B_12_F_11_]^−^ is presented. The HOMO shows increased electron delocalization over the coordinated boron atoms, while the LUMO predominantly localizes on the NH_3_ molecule. More detailed results of the molecular orbitals (MOs) of [B_12_X_11_]^−^ (X = H, F, and CN) are given in Figure S2e.

## IV Conclusions

Using density functional theory, we carried out a comprehensive analysis of the interactions of [B_12_X_12_]^2–^, [B_12_X_11_]^−^, and [B_12_X_11_]^2–^ clusters (X = H, F, and CN) with various small molecules (H_2_, O_2_, CO, NH_3_). We studied (i) the thermodynamic and dynamical stability of these systems, (ii) the strength of their binding, (iii) structural characteristics such as bond lengths and angles, and (iv) electronic properties, including charge transfer and molecular orbital analyses. In both [B_12_X_11_]^−^ monoanion and [B_12_X_11_]^2–^ dianion clusters, the “naked” boron atom consistently emerges as the most reactive site for molecule adsorption, leading to the formation of stable bonds. The results are summarized in Table 3. Molecules such as H_2_, CO, and NH_3_ bind more strongly to [B_12_X_11_]^−^ clusters than to [B_12_X_11_]^2–^ clusters while the binding of O_2_ with [B_12_X_11_]^2–^ dianion clusters is stronger compared to their monoanion counterparts. This is due to the fact that [B_12_X_11_]^2–^ has a radical character. With the exception of [B_12_X_11_]^−^ interacting with CO and NH_3_ and [B_12_X_11_]^2–^ (X = F, CN) interacting with O_2_, the binding energies of the molecules are intermediate between physisorption and chemisorption. The interaction of these molecules with the corresponding clusters leads to noticeable structural deformations such as bond elongation and molecular bending. These changes indicate the potential of B_12_X_11_ clusters in activating these molecules. These insights into the reactivity and stability of [B_12_X_11_]^−^ and [B_12_X_11_]^2–^ clusters interacting with small molecules may motivate future work on their smaller cousins such as mono- and dianionic [B*_n_*X_*n*–1_] (*n* = 6, 8, and 10).

**Table 3:** Binding Energies of H_2_, O_2_, CO, and NH_3_ Interacting with [B_12_X_11_]^−^ and [B_12_X_11_]^2–^ (X = H, F, CN)^a^

		adsorbed molecules			
	clusters	H_2_	O_2_	CO	NH_3_
binding energies (eV)	[B_12_H_11_]^−^	0.52	0.58	2.05	2.07
	[B_12_F_11_]^−^	1.33	1.63	3.00	3.42
	[B_12_(CN)_11_]^−^	1.28	1.21	2.76	4.21
	[B_12_F_11_]^2–^	not bound	2.75	1.49	0.95
	[B_12_(CN)_11_]^2–^	dissociated	2.70	1.35	0.60
bond lengths (Å)	[B_12_H_11_]^−^	0.834	1.296	1.148	1.020
	[B_12_F_11_]^−^	0.883	1.307	1.145	1.023
	[B_12_(CN)_11_]^−^	0.827	1.254	1.129	1.027
	[B_12_F_11_]^2–^	not bound	1.342	1.200	1.036
	[B_12_(CN)_11_]^2–^	dissociated	1.341	1.190	1.026

a The corresponding bond lengths of isolated H_2_, O_2_, CO, and NH_3_ molecules are 0.743, 1.215, 1.137, 0.980, and 1.016 Å, respectively. The bond length, 1.016 Å, in NH_3_ represents the N–H bond.

## Supporting Information Available

Energy cost to detach the ligand in neutral or anionic form; molecular orbitals analysis; the Cartesian coordinates, total energies, and frequencies for the lowest energetic configurations (PDF)

## References

[ref1] Longuet-Higgins H. C.; Roberts M. D. V. The Electronic Structure of an Icosahedron of Boron Atoms. Proc. R. Soc. Lond. A: Math. Phys. Sci. 1955, 230 (1180), 110–119. 10.1098/rspa.1955.0115.

[ref2] Pitochelli A. R.; Hawthorne F. M. The Isolation of the Icosahedral B_12_H_12_^2–^ ion. J. Am. Chem. Soc. 1960, 82 (12), 3228–3229. 10.1021/ja01497a069.

[ref3] Wunderlich J. A.; Lipscomb W. N. Structure of B_12_H_12_^2–^ Ion. J. Am. Chem. Soc. 1960, 82 (16), 4427–4428. 10.1021/ja01501a076.

[ref4] Warneke J.; Rohdenburg M.; Liu J. K. Y.; Johnson E.; Ma X.; Kumar R.; Su P.; Aprà E.; Wang X.-B.; Jenne C.; et al. Gas Phase Fragmentation of Adducts between Dioxygen and Closo-Borate Radical Anions. Int. J. Mass Spectrom. 2019, 436, 71–78. 10.1016/j.ijms.2018.11.005.

[ref5] Zhong M.; Zhou J.; Fang H.; Jena P. Role of Ligands in the Stability of B_n_ X_n_ and CB_n–1_ X_n_ (n = 5–10; X = H, F, CN) and Their Potential as Building Blocks of Electrolytes in Lithium-Ion Batteries. Phys. Chem. Chem. Phys. 2017, 19 (27), 17937–17943. 10.1039/C7CP02642K. 28664958

[ref6] Bolli C.; Derendorf J.; Keßler M.; Knapp C.; Scherer H.; Schulz C.; Warneke J. Synthesis, Crystal Structure, and Reactivity of the Strong Methylating Agent Me_2_B_12_Cl_12_. Angew. Chem., Int. Ed. 2010, 49 (20), 3536–3538. 10.1002/anie.200906627. 20544906

[ref7] Sivaev I. B.; Bregadze V. I.; Sjöberg S. Chemistry of Closo-Dodecaborate Anion [B12H12]2-: A Review. Collect. Czech. Chem. Commun. 2002, 67 (6), 679–727. 10.1135/cccc20020679.

[ref8] Hopkins W. S.; Carr P. J. J.; Huang D.; Bishop K. P.; Burt M.; McMahon T. B.; Steinmetz V.; Fillion E. Infrared-Driven Charge Transfer in Transition Metal B_12_F_12_ Clusters. J. Phys. Chem. A 2015, 119 (31), 8469–8475. 10.1021/acs.jpca.5b03932. 26090930

[ref9] Moon J.; Baek H.; Kim J. Unusually High Stability of B_12_(BO)_12_^2–^ Achieved by Boronyl Ligand Manipulation: Theoretical Investigation. Chem. Phys. Lett. 2018, 698, 72–76. 10.1016/j.cplett.2018.03.015.

[ref10] Warneke J.; Hou G.-L.; Aprà E.; Jenne C.; Yang Z.; Qin Z.; Kowalski K.; Wang X.-B.; Xantheas S. S. Electronic Structure and Stability of [B_12_X_12_]^2–^ (X = F–At): A Combined Photoelectron Spectroscopic and Theoretical Study. J. Am. Chem. Soc. 2017, 139 (41), 14749–14756. 10.1021/jacs.7b08598. 28933868

[ref11] Warneke J.; Dülcks T.; Knapp C.; Gabel D. Collision-Induced Gas-Phase Reactions of Perhalogenated Closo-Dodecaborate Clusters – a Comparative Study. Phys. Chem. Chem. Phys. 2011, 13 (13), 5712 10.1039/c0cp02386h. 21311775

[ref12] Barba-Bon A.; Salluce G.; Lostalé-Seijo I.; Assaf K. I.; Hennig A.; Montenegro J.; Nau W. M. Boron Clusters as Broadband Membrane Carriers. Nature 2022, 603 (7902), 637–642. 10.1038/s41586-022-04413-w. 35322251 PMC8942850

[ref13] Boeré R. T.; Derendorf J.; Jenne C.; Kacprzak S.; Keßler M.; Riebau R.; Riedel S.; Roemmele T. L.; Rühle M.; Scherer H.; et al. On the Oxidation of the Three-Dimensional Aromatics [B_12_X_12_]^2–^ (X = F, Cl, Br, I). Chem. – Eur. J. 2014, 20 (15), 4447–4459. 10.1002/chem.201304405. 24595990

[ref14] Wade K. The Structural Significance of the Number of Skeletal Bonding Electron-Pairs in Carboranes, the Higher Boranes and Borane Anions, and Various Transition-Metal Carbonyl Cluster Compounds. J. Chem. Soc. D: Chem. Commun. 1971, (15), 792 10.1039/c29710000792.

[ref15] Mingos D. M. P. Polyhedral Skeletal Electron Pair Approach. Acc. Chem. Res. 1984, 17 (9), 311–319. 10.1021/ar00105a003.

[ref16] Jemmis E. D.; Balakrishnarajan M. M.; Pancharatna P. D. A Unifying Electron-Counting Rule for Macropolyhedral Boranes, Metallaboranes, and Metallocenes. J. Am. Chem. Soc. 2001, 123 (18), 4313–4323. 10.1021/ja003233z. 11457198

[ref17] Li S.; Willis M.; Jena P. Reaction Intermediates during the Dehydrogenation of Metal Borohydrides: A Cluster Perspective. J. Phys. Chem. C 2010, 114 (39), 16849–16854. 10.1021/jp106638u.

[ref18] McKee M. L.; Wang Z.-X.; von Schleyer P. R. Ab Initio Study of the *Hypercloso* Boron Hydrides B_*n*_H_*n*_ and B_*n*_H_*n*_^–^. Exceptional Stability of Neutral B_13_H_13_. J. Am. Chem. Soc. 2000, 122 (19), 4781–4793. 10.1021/ja994490a.

[ref19] Aprà E.; Warneke J.; Xantheas S. S.; Wang X.-B. A Benchmark Photoelectron Spectroscopic and Theoretical Study of the Electronic Stability of [B_12_H_12_]^2–^. J. Chem. Phys. 2019, 150 (16), 164306 10.1063/1.5089510. 31042907

[ref20] Zhao H.; Zhou J.; Jena P. Stability of B_12_(CN)_12_^2–^ : Implications for Lithium and Magnesium Ion Batteries. Angew. Chem., Int. Ed. 2016, 55 (11), 3704–3708. 10.1002/anie.201600275. 26880349

[ref21] Mayer M.; van Lessen V.; Rohdenburg M.; Hou G.-L.; Yang Z.; Exner R. M.; Aprà E.; Azov V. A.; Grabowsky S.; Xantheas S. S. J.; et al. Rational Design of an Argon-Binding Superelectrophilic Anion. Proc. Natl. Acad. Sci. U.S.A. 2019, 116 (17), 8167–8172. 10.1073/pnas.1820812116. 30952786 PMC6486711

[ref22] Rohdenburg M.; Mayer M.; Grellmann M.; Jenne C.; Borrmann T.; Kleemiss F.; Azov V. A.; Asmis K. R.; Grabowsky S.; Warneke J. Superelectrophilic Behavior of an Anion Demonstrated by the Spontaneous Binding of Noble Gases to [B _12_Cl_11_]^−^. Angew. Chem., Int. Ed. 2017, 56 (27), 7980–7985. 10.1002/anie.201702237. 28560843

[ref23] Mayer M.; Rohdenburg M.; Kawa S.; Horn F.; Knorke H.; Jenne C.; Tonner R.; Asmis K. R.; Warneke J. Relevance of Π-Backbonding for the Reactivity of Electrophilic Anions [B_12_X_11_]^−^ (X = F, Cl, Br, I, CN). Chem. – Eur. J. 2021, 27 (40), 10274–10281. 10.1002/chem.202100949. 34014012 PMC8362024

[ref24] Kilic M. E.; Jena P. Catalytic Potential of [B_12_*X*_11_]^2–^ (*X* = F, Cl, Br, I, CN) Dianions. J. Phys. Chem. Lett. 2023, 14 (39), 8697–8701. 10.1021/acs.jpclett.3c02222. 37733639

[ref25] Lee C.; Yang W.; Parr R. G. Development of the Colle-Salvetti Correlation-Energy Formula into a Functional of the Electron Density. Phys. Rev. B 1988, 37 (2), 785–789. 10.1103/PhysRevB.37.785. 9944570

[ref26] Frisch M. J.; Trucks G. W.; Schlegel H. B.; Scuseria G. E.; Robb M. A.; Cheeseman J. R.; Scalmani G.; Barone V.; Petersson G. A.; Nakatsuji H. Gaussian 16, Revision C.01; Gaussian Inc.: Wallingford, CT, 2016.

[ref27] Wulf T.; Warneke J.; Heine T. B_12_X_11_ (H_2_)^−^ : Exploring the Limits of Isotopologue Selectivity of Hydrogen Adsorption. RSC Adv. 2021, 11 (46), 28466–28475. 10.1039/D1RA06322G. 35478551 PMC9038111

[ref28] Kang L.; Wang B.; Bing Q.; Zalibera M.; Büchel R.; Xu R.; Wang Q.; Liu Y.; Gianolio D.; Tang C. C.; et al. Adsorption and Activation of Molecular Oxygen over Atomic Copper(I/II) Site on Ceria. Nat. Commun. 2020, 11 (1), 4008 10.1038/s41467-020-17852-8. 32782245 PMC7419315

[ref29] Asmis K. R.; Beele B. B.; Jenne C.; Kawa S.; Knorke H.; Nierstenhöfer M. C.; Wang X.; Warneke J.; Warneke Z.; Yuan Q. Synthesis, Electronic Properties and Reactivity of [B_12_X_11_(NO_2_)]^2–^ (X = F–I) Dianions. Chem. – Eur. J. 2020, 26 (64), 14594–14601. 10.1002/chem.202003537. 33017100 PMC7756457

[ref30] Fielicke A.; von Helden G.; Meijer G.; Pedersen D. B.; Simard B.; Rayner D. M. Size and Charge Effects on the Binding of CO to Late Transition Metal Clusters. J. Chem. Phys. 2006, 124 (19), 194305 10.1063/1.2196887. 16729812

[ref31] Hertler W. R.; Raasch M. S. Chemistry of Boranes. XIV. Amination of B_10_H_10_^2–^ and B_12_H_12_^2–^ with Hydroxylamine-O-Sulfonic Acid. J. Am. Chem. Soc. 1964, 86 (18), 3661–3668. 10.1021/ja01072a014.

[ref32] Axtell J. C.; Saleh L. M. A.; Qian E. A.; Wixtrom A. I.; Spokoyny A. M. Synthesis and Applications of Perfunctionalized Boron Clusters. Inorg. Chem. 2018, 57 (5), 2333–2350. 10.1021/acs.inorgchem.7b02912. 29465227 PMC5985200

[ref33] Miller H. C.; Hertler W. R.; Muetterties E. L.; Knoth W. H.; Miller N. E. Chemistry of Boranes. XXV. Synthesis Andc Chemistry of Base Derivatives of B_10_H_10_^2–^ and B_12_H_12_^2–^. Inorg. Chem. 1965, 4 (8), 1216–1221. 10.1021/ic50030a028.

[ref34] Bukovsky E. V.; Pluntze A. M.; Strauss S. H. Efficient Direct Fluorination of the B12H11(NH3)– Anion in Acetonitrile and Comparison of the Structures of Na(H2O)4(B12F11(NH3)), Na(H3O)(H2O)3(B12F12), and Na2(H2O)4(B12F12). J. Fluorine Chem. 2017, 203, 90–98. 10.1016/j.jfluchem.2017.06.006.

[ref35] Borodin D.; Galparsoro O.; Rahinov I.; Fingerhut J.; Schwarzer M.; Hörandl S.; Auerbach D. J.; Kandratsenka A.; Schwarzer D.; Kitsopoulos T. N.; Wodtke A. M. Steric Hindrance of NH _3_ Diffusion on Pt(111) by Co-Adsorbed O-Atoms. J. Am. Chem. Soc. 2022, 144 (47), 21791–21799. 10.1021/jacs.2c10458. 36399044 PMC9716551

