# Supplementary material for: Activation of Small Molecules by Modified Dodecaborate Anions
Source: J Phys Chem A. 2024 Mar 8;128(11):1993–2002. doi: 10.1021/acs.jpca.3c07361 (PMC10961843; doi:10.1021/acs.jpca.3c07361)
Supplement: Supplementary file 1 — jp3c07361_si_001.pdf [file jp3c07361_si_001.pdf]

Supporting Information:

Activation of Small Molecules by Modified Dodecaborate Anions

Mehmet Emin Kilic and Puru Jena*

Department of Physics, Virginia Commonwealth University, Richmond, VA 23284-2000, USA

*Email: pjena@vcu.edu

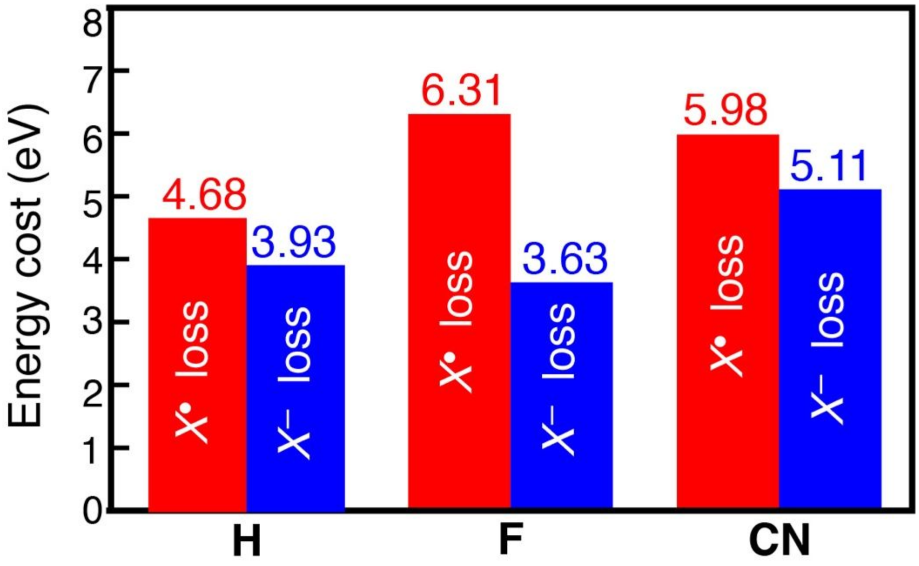

Figure S1 Energy cost to detach the ligand in neutral ($X^{\bullet}$, red pillar) or anionic form (X^{-}, blue pillar).

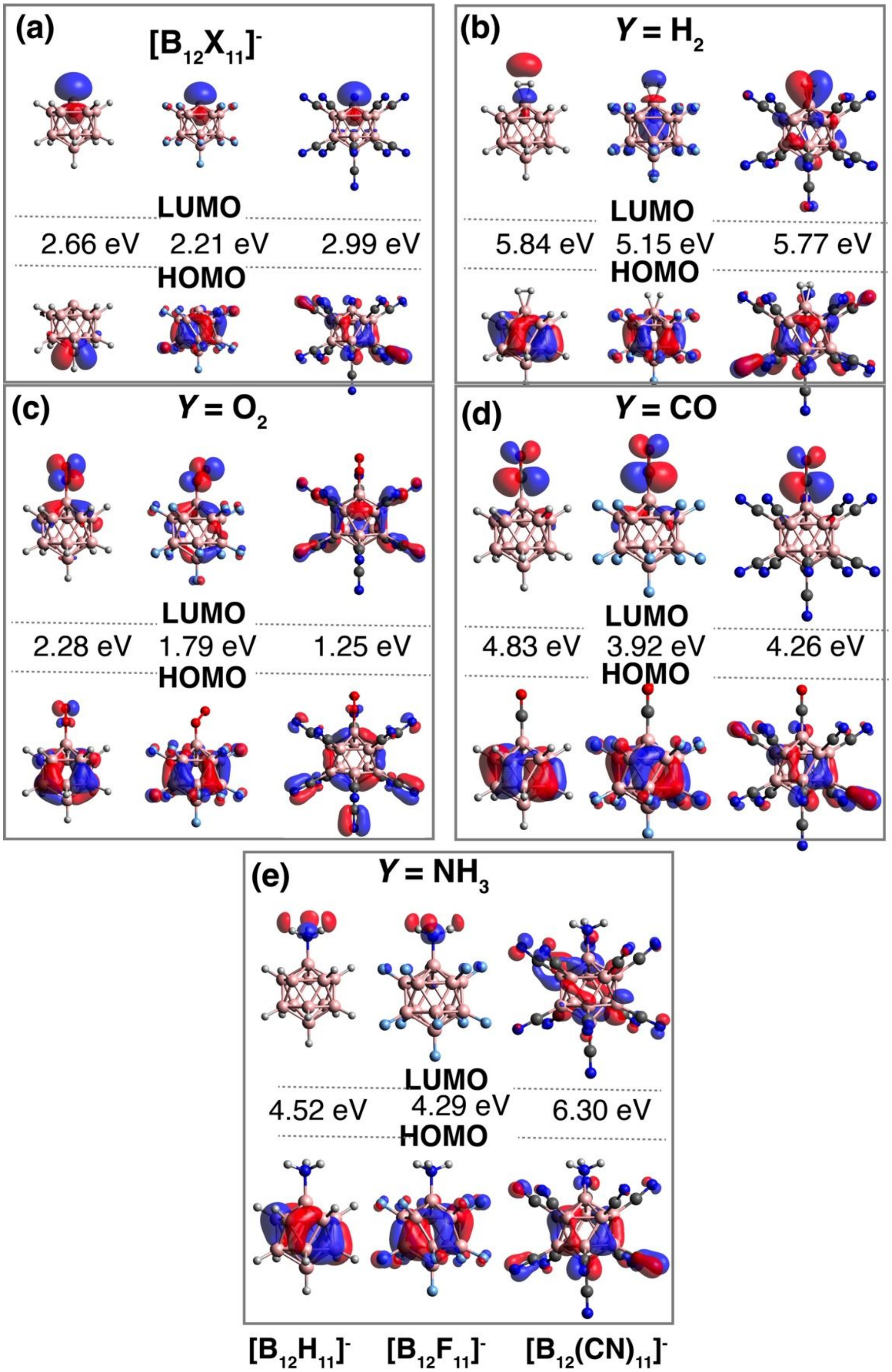

Figure S2 Molecular Orbitals Analysis of $[B_{12}X_{11}]^-$ and $[B_{12}X_{11}Y]^-$ where X = H, F, and CN ligands and $Y = H_2$, O_2, CO, and NH_3. Featuring HOMO and LUMO energy gaps and electron distributions. Blue and red shaded regions indicate positive and negative charges, respectively.

Table S1 The cartesian *xyz* coordinates in the unit of Å for the lowest energetic configurations of $[B_{12}X_{12}]^{2-}$, $[B_{12}X_{11}]^{-}$, $[B_{12}X_{11}]^{2-}$, $[B_{12}X_{11}Y]^{-}$, and $[B_{12}X_{11}Y]^{2-}$ where X = H, F, and CN, Y = H_2, O_2, CO, and NH_3.

$[B_{12}H_{12}]^{2-}$					$[B_{12}H_{1}]^{-}$					$[B_{12}H_{11}]^{2-}$			
B	-1.1148	-0.6867	1.0794		B	0.00	0.00	1.61		B	0.000	0.000	1.649
B	-1.0037	-1.2198	-0.6197		B	1.53	-0.10	0.70		B	0.012	-1.519	0.717
B	-1.6311	0.4053	-0.2338		B	0.38	-1.48	0.70		B	-1.441	-0.480	0.717
B	-0.6933	1.0459	1.1423		B	-1.29	-0.82	0.70		B	-0.902	1.223	0.716
B	0.5137	-0.1833	1.6068		B	-1.18	0.97	0.70		B	0.884	1.236	0.716
B	0.3218	-1.5835	0.5179		B	0.56	1.42	0.70		B	1.449	-0.458	0.717
B	-0.5136	0.1833	-1.6068		B	1.20	-0.99	-0.81		B	-0.885	-1.238	-0.805
B	-0.3218	1.5835	-0.5179		B	-0.57	-1.45	-0.81		B	-1.451	0.459	-0.805
B	1.0037	1.2198	0.6197		B	-1.55	0.10	-0.81		B	-0.012	1.522	-0.805
B	1.6311	-0.4053	0.2338		B	-0.39	1.51	-0.81		B	1.444	0.481	-0.805
B	0.6933	-1.0459	-1.1423		B	1.31	0.83	-0.81		B	0.904	-1.225	-0.805
B	1.1148	0.6867	-1.0794		B	0.00	0.00	-1.48		B	0.000	0.000	-1.696
H	-1.9062	-1.1742	1.8456		H	0.00	0.00	2.80		H	0.000	0.001	2.853
H	-1.7163	-2.0857	-1.0596		H	2.59	-0.16	1.23		H	0.020	-2.596	1.256
H	-2.7891	0.6930	-0.3998		H	0.65	-2.52	1.23		H	-2.463	-0.821	1.255
H	-1.1856	1.7884	1.9532		H	-2.19	-1.39	1.23		H	-1.542	2.089	1.255
H	0.8783	-0.3134	2.7476		H	-2.00	1.66	1.23		H	1.510	2.113	1.255
H	0.5502	-2.7077	0.8855		H	0.96	2.42	1.23		H	2.476	-0.783	1.255
H	-0.8783	0.3134	-2.7476		H	1.99	-1.65	-1.40		H	-1.514	-2.117	-1.336
H	-0.5502	2.7077	-0.8855		H	-0.95	-2.41	-1.40		H	-2.481	0.785	-1.337
H	1.7163	2.0857	1.0596		H	-2.58	0.16	-1.40		H	-0.020	2.602	-1.338
H	2.7891	-0.6930	0.3998		H	-0.64	2.51	-1.40		H	2.469	0.823	-1.337
H	1.1856	-1.7884	-1.9532		H	2.19	1.39	-1.40		H	1.545	-2.094	-1.337
H	1.9063	1.1741	-1.8456										

$[B_{12}H_{12}H_2]^-$			
B	1.712	0.000	0.011
B	0.775	0.899	1.235
B	0.796	1.451	-0.463
B	0.788	0.000	-1.518
B	0.796	-1.451	-0.463
B	0.775	-0.899	1.235
B	-0.715	1.473	0.465
B	-0.710	0.917	-1.246
B	-0.710	-0.917	-1.246
B	-0.715	-1.473	0.465
B	-0.732	0.000	1.536
B	-1.527	0.000	-0.011
H	2.906	0.000	0.019
H	1.294	1.523	2.111
H	1.333	2.465	-0.794
H	1.314	0.000	-2.589
H	1.333	-2.465	-0.794
H	1.294	-1.523	2.111
H	-1.315	2.454	0.787
H	-1.294	1.519	-2.095
H	-1.294	-1.519	-2.095
H	-1.315	-2.454	0.787
H	-1.324	0.000	2.571
H	-2.801	0.000	-0.430
H	-2.802	0.000	0.405

$[B_{12}H_{12}O_2]^-$			
B	2.268	0.244	0.000
B	1.389	-0.355	1.448
B	1.179	1.359	0.879
B	1.179	1.359	-0.879
B	1.389	-0.355	-1.448
B	1.595	-1.385	0.000
B	-0.191	0.413	1.472
B	-0.358	1.444	0.000
B	-0.191	0.413	-1.472
B	0.043	-1.361	-0.873
B	0.043	-1.361	0.872
B	-1.027	-0.205	0.000
H	3.448	0.414	0.000
H	1.913	-0.628	2.485
H	1.575	2.282	1.521
H	1.575	2.283	-1.521
H	1.913	-0.627	-2.485
H	2.288	-2.353	0.000
H	-0.877	0.594	2.427
H	-1.075	2.391	0.000
H	-0.877	0.594	-2.427
H	-0.428	-2.249	-1.509
H	-0.428	-2.249	1.509
O	-3.314	0.359	0.000
O	-2.389	-0.547	0.000

$[B_{12}H_{12}CO]^-$			
B	-2.260	-0.003	0.001
B	-1.331	-1.120	1.036
B	-1.332	-1.331	-0.744
B	-1.334	0.295	-1.494
B	-1.336	1.512	-0.179
B	-1.334	0.638	1.384
B	0.167	-1.535	0.182
B	0.165	-0.647	-1.405
B	0.163	1.137	-1.051
B	0.163	1.351	0.755
B	0.166	-0.300	1.517
B	1.029	0.003	-0.001
H	-3.454	-0.004	0.001
H	-1.852	-1.908	1.764
H	-1.853	-2.268	-1.267
H	-1.858	0.503	-2.546
H	-1.859	2.577	-0.306
H	-1.856	1.087	2.358
H	0.758	-2.563	0.304
H	0.754	-1.081	-2.346
H	0.750	1.898	-1.755
H	0.751	2.256	1.261
H	0.756	-0.501	2.533
C	2.511	0.001	0.000
O	3.659	0.000	0.000

$[B_{12}H_{12}NH3]^-$			
B	-2.0507	0.0009	-0.0006
B	-1.1248	-1.5208	0.0887
B	-1.1238	-0.5544	-1.4203
B	-1.1231	1.1790	-0.9668
B	-1.1236	1.2835	0.8222
B	-1.1250	-0.3852	1.4749
B	0.3825	-1.2849	-0.8235
B	0.3818	0.3854	-1.4756
B	0.3843	1.5227	-0.0889
B	0.3812	0.5545	1.4214
B	0.3814	-1.1802	0.9680
B	1.2303	-0.0010	0.0007
H	-3.2458	0.0016	-0.0011
H	-1.6566	-2.5895	0.1505
H	-1.6558	-0.9448	-2.4169
H	-1.6540	2.0083	-1.6445
H	-1.6547	2.1860	1.3986
H	-1.6577	-0.6564	2.5100
H	0.9859	-2.1604	-1.3825
H	0.9943	0.6478	-2.4764
H	0.9863	2.5602	-0.1490
H	0.9913	0.9328	2.3856
H	0.9866	-1.9842	1.6245
N	2.8186	-0.0001	0.0002
H	3.1654	0.5867	0.7590
H	3.1678	-0.9496	0.1289
H	3.1638	0.3637	-0.8881

$[B_{12}F_{12}]^{2-}$				$[B_{12}F_{11}]^{-}$				$[B_{12}F_{11}]^{2-}$			
B	1.398	-0.116	0.969	B	0.000	0.000	1.523	B	0.000	0.000	1.513
B	0.288	1.288	1.079	B	-0.382	-1.492	0.595	B	0.428	-1.470	0.580
B	1.287	1.048	-0.390	B	-1.537	-0.098	0.595	B	-1.265	-0.862	0.580
B	1.371	-0.712	-0.721	B	-0.568	1.432	0.595	B	-1.211	0.937	0.580
B	0.424	-1.559	0.544	B	1.186	0.982	0.595	B	0.517	1.441	0.580
B	-0.245	-0.323	1.656	B	1.301	-0.824	0.595	B	1.530	-0.047	0.581
B	-0.424	1.559	-0.544	B	-1.213	-1.005	-0.923	B	-0.516	-1.438	-0.944
B	0.245	0.323	-1.656	B	-1.330	0.843	-0.923	B	-1.527	0.047	-0.944
B	-0.288	-1.288	-1.079	B	0.391	1.526	-0.923	B	-0.427	1.467	-0.944
B	-1.287	-1.048	0.390	B	1.572	0.100	-0.923	B	1.263	0.860	-0.944
B	-1.371	0.712	0.721	B	0.581	-1.464	-0.924	B	1.209	-0.936	-0.944
B	-1.398	0.116	-0.969	B	0.000	0.000	-1.646	B	0.000	0.000	-1.799
F	2.542	-0.210	1.763	F	0.000	0.000	2.900	F	0.000	0.000	2.908
F	0.524	2.342	1.962	F	-0.688	-2.685	1.207	F	0.775	-2.661	1.221
F	2.341	1.905	-0.709	F	-2.766	-0.176	1.207	F	-2.291	-1.560	1.220
F	2.493	-1.295	-1.311	F	-1.022	2.576	1.207	F	-2.192	1.697	1.220
F	0.771	-2.836	0.988	F	2.134	1.768	1.207	F	0.936	2.608	1.221
F	-0.446	-0.588	3.011	F	2.341	-1.484	1.207	F	2.770	-0.085	1.221
F	-0.771	2.836	-0.988	F	-2.136	-1.769	-1.591	F	-0.940	-2.617	-1.568
F	0.446	0.588	-3.011	F	-2.342	1.484	-1.591	F	-2.779	0.085	-1.569
F	-0.524	-2.342	-1.962	F	0.688	2.686	-1.591	F	-0.778	2.670	-1.568
F	-2.341	-1.905	0.709	F	2.767	0.176	-1.591	F	2.299	1.565	-1.568
F	-2.493	1.295	1.311	F	1.022	-2.577	-1.591	F	2.199	-1.703	-1.568
F	-2.542	0.210	-1.763								

$[B_{12}F_{11}H_2]^-$					$[B_{12}F_{11}O_2]^-$					$[B_{12}F_{11}O_2]^{2-}$			
B	0.001	0.001	1.568		B	1.794	0.470	0.001		B	-1.814	-0.415	0.000
B	-1.277	0.856	0.627		B	0.731	0.678	1.465		B	-0.786	-0.640	1.451
B	0.411	1.487	0.643		B	0.489	1.710	0.003		B	-0.525	-1.663	0.002
B	1.536	0.068	0.620		B	0.731	0.682	-1.463		B	-0.786	-0.644	-1.449
B	0.533	-1.446	0.641		B	1.245	-1.014	-0.887		B	-1.203	1.012	-0.899
B	-1.200	-0.963	0.634		B	1.245	-1.016	0.884		B	-1.203	1.014	0.896
B	-0.530	1.465	-0.875		B	-0.917	1.045	0.884		B	0.884	-1.015	0.902
B	1.205	0.983	-0.879		B	-0.918	1.047	-0.880		B	0.884	-1.017	-0.900
B	1.284	-0.874	-0.880		B	-0.406	-0.689	-1.475		B	0.459	0.642	-1.455
B	-0.411	-1.504	-0.872		B	-0.141	-1.707	-0.003		B	0.203	1.665	-0.002
B	-1.550	-0.072	-0.880		B	-0.406	-0.694	1.473		B	0.459	0.645	1.454
B	-0.001	0.000	-1.728		B	-1.446	-0.442	-0.001		B	1.478	0.410	0.000
F	0.003	0.001	2.942		F	3.103	0.842	0.001		F	-3.165	-0.756	0.001
F	-2.312	1.528	1.235		F	1.214	1.189	2.635		F	-1.295	-1.167	2.636
F	0.746	2.666	1.269		F	0.792	3.050	0.005		F	-0.827	-3.023	0.004
F	2.772	0.117	1.221		F	1.214	1.197	-2.632		F	-1.294	-1.173	-2.633
F	0.973	-2.592	1.264		F	2.119	-1.764	-1.628		F	-2.054	1.838	-1.630
F	-2.173	-1.717	1.248		F	2.119	-1.769	1.623		F	-2.054	1.841	1.626
F	-0.962	2.582	-1.558		F	-1.795	1.763	1.642		F	1.705	-1.841	1.656
F	2.156	1.717	-1.553		F	-1.795	1.768	-1.637		F	1.705	-1.845	-1.651
F	2.295	-1.528	-1.547		F	-0.925	-1.191	-2.626		F	0.972	1.167	-2.637
F	-0.739	-2.654	-1.560		F	-0.456	-3.044	-0.005		F	0.508	3.024	-0.004
F	-2.757	-0.120	-1.539		F	-0.925	-1.199	2.622		F	0.971	1.173	2.634
H	0.433	-0.039	-2.944		O	-2.753	-0.919	-0.001		O	2.853	0.906	-0.001
H	-0.449	0.032	-2.938		O	-3.746	-0.071	0.000		O	3.799	-0.044	0.000

$[B_{12}F_{11}CO]^-$					$[B_{12}F_{11}CO]^{2-}$				
B	-1.847	0.000	0.000		B	**-1.806**	0.000	-0.365	
B	-0.910	-0.532	1.446		B	-0.780	1.451	-0.623	
B	-0.910	-1.540	-0.059		B	-1.158	0.896	1.046	
B	-0.910	-0.420	-1.482		B	-1.158	-0.896	1.046	
B	-0.910	1.280	-0.857		B	-0.780	-1.451	-0.623	
B	-0.910	1.211	0.953		B	-0.563	0.000	-1.656	
B	0.598	-1.294	0.866		B	0.500	1.447	0.634	
B	0.598	-1.224	-0.963		B	0.261	0.000	1.663	
B	0.598	0.538	-1.461		B	0.500	-1.447	0.634	
B	0.598	1.556	0.060		B	0.866	-0.894	-1.046	
B	0.598	0.424	1.498		B	0.866	0.894	-1.046	
B	1.486	0.000	0.000		B	1.503	0.000	0.368	
F	-3.218	0.000	0.000		F	-3.165	0.000	-0.669	
F	-1.524	-0.956	2.600		F	-1.305	2.639	-1.126	
F	-1.524	-2.768	-0.106		F	-1.987	1.633	1.889	
F	-1.525	-0.754	-2.665		F	-1.987	-1.633	1.889	
F	-1.525	2.302	-1.541		F	-1.305	-2.639	-1.126	
F	-1.524	2.177	1.713		F	-0.914	0.000	-3.004	
F	1.258	-2.295	1.537		F	1.018	2.637	1.138	
F	1.257	-2.171	-1.708		F	0.594	0.000	3.015	
F	1.257	0.954	-2.593		F	1.018	-2.637	1.138	
F	1.257	2.760	0.106		F	1.686	-1.635	-1.890	
F	1.258	0.752	2.658		F	1.686	1.635	-1.890	
C	2.960	0.000	0.000		C	3.039	0.000	0.761	
O	4.104	0.000	0.000		O	4.057	0.000	0.126	

	$[B_{12}F_{11}NH_3]^-$					$[B_{12}F_{11}NH_3]^{2-}$		
	B	-1.749	0.000	-0.024	B	1.738	0.000	0.004
	B	-0.838	0.000	1.532	B	0.804	0.001	-1.529
	B	-0.824	-1.465	0.466	B	0.801	-1.455	-0.471
	B	-0.800	-0.906	-1.259	B	0.797	-0.900	1.240
	B	-0.800	0.906	-1.259	B	0.797	0.899	1.241
	B	-0.824	1.465	0.465	B	0.802	1.455	-0.470
	B	0.679	-0.899	1.249	B	-0.714	-0.896	-1.237
	B	0.701	-1.456	-0.463	B	-0.719	-1.451	0.469
	B	0.716	0.000	-1.519	B	-0.721	-0.001	1.524
	B	0.701	1.456	-0.463	B	-0.719	1.450	0.470
	B	0.679	0.900	1.249	B	-0.714	0.897	-1.236
	B	1.551	0.000	0.023	B	-1.616	0.000	-0.004
	F	-3.129	0.000	-0.043	F	3.130	0.000	0.008
	F	-1.479	0.000	2.754	F	1.437	0.001	-2.769
	F	-1.450	-2.637	0.838	F	1.430	-2.637	-0.854
	F	-1.410	-1.632	-2.262	F	1.423	-1.631	2.246
	F	-1.410	1.631	-2.263	F	1.423	1.630	2.247
	F	-1.450	2.638	0.837	F	1.430	2.638	-0.852
	F	1.412	-1.599	2.206	F	-1.376	-1.620	-2.230
	F	1.444	-2.581	-0.814	F	-1.380	-2.621	0.846
	F	1.493	0.000	-2.678	F	-1.392	-0.001	2.748
	F	1.444	2.581	-0.815	F	-1.380	2.621	0.847
	F	1.412	1.600	2.206	F	-1.376	1.622	-2.229
	N	3.108	0.000	0.031	N	-3.180	0.000	-0.006
	H	3.461	0.831	0.512	H	-3.580	0.827	-0.484
	H	3.461	0.000	-0.929	H	-3.580	-0.001	0.949
	H	3.461	-0.830	0.513	H	-3.580	-0.826	-0.485

$[B_{12}(CN)_{12}]^{2-}$				$[B_{12}(CN)_{11}]^{-}$				$[B_{12}(CN)_{11}]^{2-}$			
B	0.453	1.070	-1.242	B	0.000	0.000	-1.656	B	0.000	0.000	-1.911
B	0.119	1.644	0.418	B	-1.157	1.061	-0.967	B	1.466	-0.392	-1.012
B	-1.215	1.027	-0.600	B	-1.367	-0.772	-0.967	B	0.826	1.273	-1.012
B	-0.590	-0.348	-1.557	B	0.312	-1.538	-0.967	B	-0.955	1.179	-1.012
B	1.131	-0.581	-1.129	B	1.560	-0.179	-0.967	B	-1.416	-0.544	-1.012
B	1.569	0.650	0.091	B	0.652	1.428	-0.967	B	0.080	-1.515	-1.012
B	-1.131	0.581	1.129	B	-1.534	0.176	0.562	B	1.421	0.546	0.517
B	-1.569	-0.650	-0.091	B	-0.641	-1.404	0.561	B	-0.080	1.521	0.517
B	-0.119	-1.644	-0.418	B	1.137	-1.044	0.561	B	-1.471	0.394	0.517
B	1.215	-1.027	0.600	B	1.344	0.759	0.561	B	-0.829	-1.277	0.517
B	0.590	0.348	1.557	B	-0.307	1.513	0.561	B	0.959	-1.183	0.517
B	-0.453	-1.070	1.242	B	0.000	0.000	1.488	B	0.000	0.000	1.453
C	0.863	2.037	-2.364	C	-2.132	1.956	-1.740	C	2.799	-0.749	-1.699
C	0.226	3.130	0.797	C	-2.520	-1.423	-1.740	C	1.577	2.430	-1.699
C	-2.314	1.956	-1.142	C	0.574	-2.836	-1.740	C	-1.824	2.251	-1.699
C	-1.123	-0.662	-2.964	C	2.875	-0.330	-1.740	C	-2.705	-1.040	-1.698
C	2.153	-1.106	-2.150	C	1.202	2.632	-1.740	C	0.152	-2.894	-1.698
C	2.987	1.238	0.174	C	-2.905	0.334	1.236	C	2.704	1.039	1.207
C	-2.153	1.106	2.150	C	-1.215	-2.659	1.236	C	-0.152	2.893	1.207
C	-2.987	-1.238	-0.174	C	2.154	-1.977	1.235	C	-2.798	0.749	1.208
C	-0.226	-3.130	-0.797	C	2.546	1.438	1.236	C	-1.577	-2.430	1.208
C	2.314	-1.956	1.142	C	-0.580	2.865	1.236	C	1.824	-2.250	1.207
C	1.123	0.662	2.964	C	0.001	0.000	3.026	C	0.000	0.000	2.991
C	-0.863	-2.037	2.364	N	3.459	1.953	1.737	N	-2.143	-3.303	1.725
N	3.144	-2.658	1.552	N	-2.862	2.627	-2.347	N	3.799	-1.017	-2.228
N	0.308	4.254	1.083	N	0.772	-3.807	-2.347	N	-2.476	3.055	-2.228
N	-1.526	-0.900	-4.027	N	-3.384	-1.910	-2.346	N	2.141	3.299	-2.228
N	-3.144	2.658	-1.552	N	1.614	3.534	-2.346	N	0.207	-3.928	-2.227
N	1.173	2.768	-3.212	N	3.860	-0.443	-2.347	N	-3.671	-1.411	-2.227
N	4.059	1.682	0.236	N	2.926	-2.686	1.736	N	-3.804	1.018	1.725
N	2.926	-1.503	-2.922	N	-0.789	3.893	1.737	N	2.480	-3.059	1.725
N	-0.308	-4.254	-1.083	N	0.001	-0.001	4.188	N	0.000	0.001	4.154
N	1.526	0.900	4.027	N	-1.651	-3.613	1.737	N	-0.207	3.933	1.724
N	-1.173	-2.768	3.212	N	-3.947	0.453	1.737	N	3.676	1.413	1.724
N	-4.059	-1.682	-0.236								
N	-2.926	1.503	2.922								

$[B_{12}(CN)_{11}H_2]^-$			
B	0.000	0.000	-1.755
B	-1.490	-0.389	-0.922
B	-0.094	-1.542	-0.914
B	1.430	-0.572	-0.922
B	0.974	1.196	-0.919
B	-0.821	1.307	-0.917
B	-0.972	-1.182	0.595
B	0.819	-1.293	0.597
B	1.481	0.383	0.588
B	0.094	1.529	0.599
B	-1.422	0.564	0.589
B	0.000	0.000	1.527
C	-2.776	-0.717	-1.687
C	-0.177	-2.858	-1.693
C	2.665	-1.054	-1.688
C	1.823	2.210	-1.692
C	-1.536	2.417	-1.692
C	-1.854	-2.238	1.267
C	1.563	-2.450	1.270
C	2.818	0.724	1.253
C	0.180	2.900	1.276
C	-2.706	1.066	1.256
C	0.001	0.001	3.059

$[B_{12}(CN)_{11}O_2]^-$			
B	1.255	0.000	-0.900
B	1.384	0.001	0.855
B	0.795	1.467	-0.022
B	-0.157	0.901	-1.447
B	-0.157	-0.902	-1.447
B	0.796	-1.466	-0.021
B	-0.023	0.903	1.463
B	-0.983	1.455	0.042
B	-1.572	-0.001	-0.831
B	-0.982	-1.455	0.043
B	-0.022	-0.902	1.463
B	-1.489	0.000	0.962
C	2.808	0.002	1.409
C	1.678	2.714	-0.100
C	-0.138	1.691	-2.759
C	-0.137	-1.692	-2.758
C	1.680	-2.713	-0.098
C	0.059	1.708	2.762
C	-1.779	2.762	0.070
C	-2.904	-0.002	-1.585
C	-1.777	-2.764	0.072
C	0.060	-1.707	2.763
C	-2.750	0.000	1.829

$[B_{12}(CN)_{11}O_2]^{2-}$			
B	-0.964	0.000	-1.370
B	-1.404	-0.896	0.111
B	-0.030	-1.448	-0.891
B	0.813	0.000	-1.516
B	-0.030	1.448	-0.891
B	-1.404	0.896	0.110
B	0.114	-1.446	0.888
B	1.483	-0.895	-0.120
B	1.483	0.895	-0.120
B	0.114	1.446	0.888
B	-0.736	0.000	1.506
B	1.049	0.000	1.365
C	-2.696	-1.721	0.228
C	-0.103	-2.748	-1.708
C	1.504	0.000	-2.890
C	-0.103	2.748	-1.708
C	-2.695	1.721	0.228
C	0.180	-2.753	1.695
C	2.788	-1.701	-0.227
C	2.788	1.701	-0.227
C	0.180	2.753	1.695
C	-1.434	0.000	2.876
C	1.958	0.000	2.604

N	0.246	3.946	1.777		N	-2.371	-3.762	0.091		N	0.232	3.741	2.304
N	-3.721	-0.956	-2.319		N	3.937	0.002	1.705		N	-3.658	-2.364	0.337
N	3.574	-1.407	-2.321		N	-0.087	2.281	-3.758		N	2.026	0.000	-3.928
N	-0.238	-3.825	-2.334		N	2.395	3.625	-0.181		N	-0.161	-3.729	-2.329
N	-2.064	3.232	-2.331		N	2.398	-3.623	-0.179		N	-3.658	2.365	0.337
N	2.447	2.954	-2.329		N	-0.086	-2.284	-3.757		N	-0.161	3.729	-2.329
N	3.837	0.984	1.747		N	-3.911	-0.002	-2.166		N	3.774	2.311	-0.310
N	-3.685	1.448	1.751		N	0.142	-2.317	3.748		N	-1.961	0.000	3.912
N	0.001	0.001	4.221		N	-3.706	-0.001	2.489		N	2.646	0.000	3.541
N	2.131	-3.332	1.769		N	-2.374	3.760	0.088		N	3.773	-2.312	-0.310
N	-2.527	-3.044	1.766		N	0.140	2.319	3.747		N	0.232	-3.741	2.304
H	-0.415	-0.005	-3.041		O	3.682	0.000	-1.211		O	-3.052	0.000	-2.389
H	0.413	-0.001	-3.042		O	2.524	0.000	-1.691		O	-1.726	0.000	-2.595

$[B_{12}(CN)_{11}CO]^-$					$[B_{12}(CN)_{11}CO]^{2-}$				
B	-1.564	0.000	0.000		B	1.162	0.000	-1.224	
B	-0.689	-1.007	1.165		B	0.172	1.440	-0.887	
B	-0.689	-1.419	-0.598		B	-0.586	0.000	-1.615	
B	-0.689	0.130	-1.534		B	0.172	-1.440	-0.887	
B	-0.689	1.500	-0.350		B	1.386	-0.889	0.313	
B	-0.689	0.797	1.318		B	1.386	0.889	0.313	
B	0.822	-1.490	0.348		B	-1.440	0.893	-0.329	
B	0.822	-0.791	-1.310		B	-1.440	-0.893	-0.329	
B	0.822	1.001	-1.158		B	-0.220	-1.446	0.860	
B	0.822	1.410	0.594		B	0.520	0.000	1.604	
B	0.822	-0.130	1.525		B	-0.221	1.447	0.860	
B	1.757	0.000	0.000		B	-1.219	0.000	1.203	
C	-1.458	-1.874	2.167		C	0.358	2.747	-1.677	
C	-1.458	-2.640	-1.113		C	-1.082	0.000	-3.071	
C	-1.458	0.243	-2.854		C	0.358	-2.747	-1.677	
C	-1.458	2.790	-0.651		C	2.657	-1.704	0.603	

C	-1.457	1.482	2.452		C	2.657	1.705	0.603
C	1.491	-2.831	0.661		C	-2.714	1.705	-0.615
C	1.491	-1.504	-2.489		C	-2.714	-1.705	-0.615
C	1.491	1.902	-2.199		C	-0.401	-2.758	1.643
C	1.492	2.679	1.129		C	1.013	0.000	3.060
C	1.492	-0.246	2.897		C	-0.402	2.758	1.643
C	3.288	0.000	0.000		C	-2.297	0.000	2.299
C	-3.089	0.000	0.000		C	2.224	0.000	-2.402
N	1.988	3.647	1.537		N	1.389	0.000	4.160
N	-2.099	-2.508	2.900		N	0.507	3.729	-2.280
N	-2.100	0.325	-3.820		N	0.507	-3.729	-2.280
N	-2.100	-3.533	-1.489		N	-1.463	0.000	-4.170
N	-2.099	1.983	3.281		N	3.612	2.329	0.824
N	-2.099	3.734	-0.871		N	3.612	-2.328	0.824
N	1.988	2.589	-2.994		N	-0.537	-3.749	2.234
N	1.988	-0.335	3.943		N	-0.538	3.749	2.233
N	4.450	0.000	-0.001		N	-3.112	0.000	3.128
N	1.987	-2.047	-3.388		N	-3.676	-2.320	-0.833
N	1.988	-3.854	0.900		N	-3.676	2.320	-0.833
O	-4.217	0.000	0.000		O	3.415	0.000	-2.402

$[B_{12}(CN)_{11}NH_3]^-$					$[B_{12}(CN)_{11}NH_3]^{2-}$			
B	-1.622	-0.106	-0.049		B	1.571	0.390	-0.024
B	-0.664	-1.433	0.604		B	0.611	0.672	1.443
B	-0.634	-1.067	-1.147		B	0.421	1.650	-0.097
B	-0.743	0.706	-1.343		B	0.609	0.500	-1.513
B	-0.841	1.437	0.286		B	1.015	-1.100	-0.805
B	-0.790	0.115	1.488		B	1.016	-0.999	0.927
B	0.880	-1.444	-0.287		B	-1.055	1.109	0.805
B	0.830	-0.114	-1.499		B	-1.056	1.008	-0.928
B	0.701	1.444	-0.606		B	-0.650	-0.675	-1.451
B	0.672	1.076	1.156		B	-0.445	-1.657	0.097
B	0.782	-0.709	1.353		B	-0.648	-0.501	1.520
B	1.706	0.111	0.052		B	-1.660	-0.408	0.025
C	-1.434	-2.663	1.103		C	1.364	1.304	2.617
C	-1.392	-1.987	-2.114		C	0.992	3.065	-0.179
C	-1.603	1.292	-2.472		C	1.361	0.991	-2.753
C	-1.760	2.645	0.521		C	2.070	-1.974	-1.494

C	-1.700	0.198	2.722	C	2.072	-1.787	1.711
C	1.643	-2.748	-0.547	C	-1.958	2.100	1.548
C	1.548	-0.219	-2.849	C	-1.960	1.905	-1.780
C	1.303	2.743	-1.153	C	-1.216	-1.307	-2.727
C	1.247	2.044	2.196	C	-0.806	-3.143	0.184
C	1.457	-1.351	2.571	C	-1.212	-0.980	2.862
C	3.235	0.211	0.098	C	-3.156	-0.762	0.047
N	1.662	2.782	2.992	N	-1.059	-4.278	0.250
N	-2.135	-3.528	1.439	N	2.057	1.794	3.419
N	-2.387	1.683	-3.236	N	2.053	1.384	-3.606
N	-2.097	-2.635	-2.774	N	1.550	4.090	-0.239
N	-2.528	0.241	3.537	N	2.959	-2.303	2.266
N	-2.560	3.474	0.676	N	2.956	-2.551	-1.987
N	1.739	3.735	-1.572	N	-1.625	-1.792	-3.703
N	1.949	-1.842	3.502	N	-1.620	-1.349	3.889
N	4.394	0.287	0.133	N	-4.290	-1.024	0.063
N	2.072	-0.300	-3.883	N	-2.625	2.597	-2.440
N	2.202	-3.747	-0.747	N	-2.621	2.864	2.125
N	-3.172	-0.205	-0.093	N	3.084	0.732	-0.045
H	-3.474	-1.129	-0.422	H	3.323	1.327	0.756
H	-3.568	0.497	-0.728	H	3.322	1.230	-0.909
H	-3.573	-0.055	0.841	H	3.651	-0.122	0.005

Table S2 Total energies in the unit of Hartree for the lowest energetic configurations of $[B_{12}X_{12}]^{2-}$, $[B_{12}X_{11}]^{-}$, $[B_{12}X_{11}]^{2-}$, $[B_{12}X_{11}Y]^{-}$, and $[B_{12}X_{11}Y]^{2-}$ where X = H, F, and CN, Y = H_2, O_2, CO, and NH_3.

	Energy (in Hartree)
**$[B_{12}H_{12}]^{2-}$**	-305.793
**$[B_{12}H_{11}]^{-}$**	-305.185
**$[B_{12}H_{11}H_2]^{-}$**	-306.383
**$[B_{12}H_{11}O_2]^{-}$**	-455.534
**$[B_{12}H_{11}CO]^{-}$**	-418.578
**$[B_{12}H_{11}NH_3]^{-}$**	-361.829

	Energy (in Hartree)			Energy (in Hartree)
**$[B_{12}F_{12}]^{2-}$**	-1497.400		**$[B_{12}(CN)_{12}]^{2-}$**	-1413.208
**$[B_{12}F_{11}]^{-}$**	-1397.404		**$[B_{12}(CN)_{11}]^{-}$**	-1320.142
**$[B_{12}F_{11}]^{2-}$**	-1397.435		**$[B_{12}(CN)_{11}]^{2-}$**	-1320.259
**$[B_{12}F_{11}H_2]^{-}$**	-1398.632		**$[B_{12}(CN)_{11}H_2]^{-}$**	-1321.367
**$[B_{12}F_{11}O_2]^{-}$**	-1547.792		**$[B_{12}(CN)_{11}O_2]^{-}$ (triplet)**	-1470.514
**$[B_{12}F_{11}O_2]^{2-}$**	-1547.864		**$[B_{12}(CN)_{11}O_2]^{2-}$**	-1470.686
**$[B_{12}F_{11}CO]^{-}$**	-1510.832		**$[B_{12}(CN)_{11}CO]^{-}$**	-1433.561
**$[B_{12}F_{11}CO]^{2-}$**	-1510.808		**$[B_{12}(CN)_{11}CO]^{2-}$**	-1433.626
**$[B_{12}F_{11}NH_3]^{-}$**	-1454.098		**$[B_{12}(CN)_{11}NH_3]^{-}$**	-1376.865
**$[B_{12}F_{11}\ NH_3]^{2-}$**	-1453.972		**$[B_{12}(CN)_{11}NH_3]^{2-}$**	-1376.849

Table S3 Vibrational frequencies in the unit of cm^{-1} for the lowest energetic configurations of $[B_{12}X_{12}]^{2-}$, $[B_{12}X_{11}]^{-}$, $[B_{12}X_{11}]^{2-}$, $[B_{12}X_{11}Y]^{-}$, and $[B_{12}X_{11}Y]^{2-}$ where X = H, F, and CN, Y = H_2, O_2, CO, and NH_3.

**$[B_{12}H_{12}]^{2-}$**	**$[B_{12}H_{11}]^{-}$**	**$[B_{12}H_{11}]^{2-}$**	**$[B_{12}H_{11}H_2]^{-}$**	**$[B_{12}H_{11}O_2]^{-}$**	**$[B_{12}H_{11}CO]^{-}$**	**$[B_{12}H_{11}NH_3]^{-}$**
522.55	493.09	510.54	35.50	38.07	76.24	35.74
522.74	493.69	510.86	418.51	165.73	76.84	184.17
522.88	495.29	518.57	508.71	180.17	361.19	189.69
523.25	495.46	525.24	510.55	326.29	489.43	428.20
523.29	510.63	525.31	522.97	447.36	489.67	521.98
576.65	545.87	564.49	523.08	454.35	507.24	523.69
576.82	545.99	576.39	526.80	488.34	507.66	523.89
577.20	553.91	576.51	540.96	511.61	534.28	528.24
577.48	554.20	576.79	564.52	526.45	563.70	528.39
577.72	567.99	576.90	568.96	544.13	563.85	577.36
661.18	645.76	656.12	577.92	545.29	566.39	577.46

661.41	646.16	656.51	588.20	556.21	566.54	582.49
661.49	654.07	665.88	607.04	557.61	582.22	582.95
661.56	654.25	666.08	649.41	571.78	582.39	636.68
711.01	692.95	710.19	654.92	583.90	628.38	662.85
711.10	703.65	712.82	660.10	602.93	630.23	662.86
711.59	710.66	712.91	670.47	630.30	630.76	667.12
745.23	711.01	741.98	703.65	633.95	666.99	667.45
748.25	718.09	742.54	722.40	668.36	667.32	717.40
748.32	718.61	742.61	730.17	682.23	715.86	722.50
748.38	748.94	746.71	733.09	688.26	727.95	722.53
748.66	749.11	753.57	737.48	718.35	728.12	744.50
761.58	750.24	753.68	741.56	725.99	737.46	744.94
762.40	759.01	756.37	745.76	726.95	737.49	748.39
762.82	759.82	756.54	756.95	732.06	737.57	756.96
762.83	759.93	759.26	759.70	736.19	752.17	757.23
762.92	770.32	759.86	760.83	741.90	752.77	761.79
762.97	770.92	759.99	764.61	746.03	759.15	761.95
763.16	800.71	823.58	764.96	754.87	759.24	765.51
763.20	800.77	823.63	767.43	759.25	765.58	767.62
869.28	860.71	859.98	768.71	762.56	765.64	768.32
869.33	860.88	859.99	853.60	771.51	771.75	778.35
869.36	884.80	882.17	854.64	774.73	841.49	779.98
869.66	884.84	882.39	857.69	795.39	841.83	858.92
942.37	914.72	929.15	870.73	816.12	859.51	859.25
942.42	919.71	932.75	900.70	816.37	859.60	887.39
942.46	919.78	932.96	900.95	848.01	900.43	887.90
942.54	923.66	940.33	935.75	862.00	900.58	889.16
948.90	923.79	940.33	939.39	878.97	912.57	936.76
948.97	930.10	944.34	942.49	896.98	929.65	937.13
949.01	932.71	944.62	946.91	907.58	929.92	942.72
949.08	932.80	944.83	948.74	916.77	941.53	942.88
949.12	940.73	946.75	951.24	923.60	941.74	947.15
952.81	940.80	946.80	952.52	925.02	948.13	947.66
953.03	943.74	948.30	953.19	929.50	948.18	949.09
953.07	943.76	948.73	954.67	936.91	948.24	951.00
953.42	955.93	958.46	954.80	943.10	951.00	951.12
953.72	970.68	958.58	958.62	943.93	951.16	957.60
963.06	971.01	960.68	958.85	951.78	953.54	957.81
963.10	1057.06	1052.91	960.25	952.81	953.68	960.54

963.19	1057.11	1053.02	961.36	960.65	958.28	960.70
1073.77	1059.89	1066.69	962.94	961.77	958.29	961.50
1073.81	2606.79	2493.91	1052.23	964.70	961.85	986.64
1074.07	2608.23	2493.95	1066.57	1013.23	1039.33	987.14
2491.98	2608.27	2497.28	1076.05	1025.50	1051.13	1008.52
2492.02	2616.07	2500.46	1174.18	1053.06	1051.27	1076.86
2492.04	2616.10	2500.47	1979.79	1114.29	1118.81	1077.50
2499.51	2627.53	2501.78	2597.32	1222.11	2159.66	1111.86
2499.51	2630.62	2501.80	2597.71	2607.91	2602.25	1334.81
2499.53	2630.65	2513.21	2597.80	2608.87	2604.39	1650.52
2499.56	2637.76	2521.03	2601.67	2618.09	2604.42	1650.81
2499.59	2637.78	2521.04	2602.28	2618.37	2610.45	2528.00
2520.95	2653.15	2553.48	2604.19	2626.66	2610.47	2528.76
2520.97			2604.19	2636.22	2615.16	2538.93
2520.99			2612.03	2641.19	2615.27	2543.91
2560.39			2616.95	2646.01	2620.09	2554.37
			2618.68	2652.31	2627.68	2583.66
			2638.65	2654.74	2627.74	2583.73
			3177.93	2665.35	2646.43	2586.15
						2595.16
						2595.21
						2618.83
						3441.87
						3559.24
						3560.58

**$[B_{12}F_{12}]^{2-}$**	**$[B_{12}F_{11}]^{-}$**	**$[B_{12}F_{11}]^{2-}$**	**$[B_{12}F_{11}O_2]^{-}$**	**$[B_{12}F_{11}O_2]^{2-}$**
173.82	82.82	177.24	76.49	45.63
173.86	83.01	177.33	106.79	112.55
173.88	145.51	177.66	125.24	159.66
173.98	145.54	177.68	168.42	174.15
178.01	166.58	178.64	168.74	174.38
178.05	166.59	178.83	171.70	176.42
178.06	170.21	179.51	172.98	176.91
178.11	172.77	179.60	173.69	177.77
178.19	172.80	180.00	174.21	178.48

179.40	172.87	180.04	175.87	179.01
179.43	172.91	180.82	176.57	179.03
179.45	177.90	180.86	177.00	179.35
179.46	184.30	186.31	181.31	179.78
179.53	184.33	186.34	182.13	181.43
188.49	184.80	188.39	183.12	181.82
188.51	184.84	188.41	185.09	187.18
188.54	220.57	227.29	185.20	187.24
188.58	220.60	227.34	189.22	189.20
234.24	232.99	243.21	189.72	190.56
234.25	356.47	384.67	218.25	222.13
234.33	356.69	384.81	225.29	231.33
386.12	368.96	389.94	230.96	232.31
386.31	369.03	390.01	301.32	295.38
386.43	401.89	400.19	349.77	384.52
386.46	441.27	440.59	351.07	384.71
386.59	470.91	464.92	389.46	387.72
423.69	470.97	465.03	389.52	388.27
473.61	506.14	508.71	392.72	404.10
473.72	506.35	509.04	428.86	430.87
473.77	529.88	540.10	452.09	473.58
545.16	543.14	555.21	466.18	473.80
545.38	543.43	617.44	472.70	475.40
545.52	569.06	617.62	504.67	538.15
633.65	594.16	626.12	533.77	544.67
633.89	594.59	627.12	552.28	562.46
633.95	601.94	627.21	561.66	626.08
634.04	602.01	706.03	579.03	631.00
634.24	608.29	706.13	590.79	631.33
726.39	681.71	750.48	611.14	635.69
726.41	681.90	754.24	618.65	647.18
726.53	719.81	754.38	639.97	710.43
755.44	719.94	766.89	643.73	723.77
755.67	747.35	767.10	664.46	735.23
755.73	747.57	768.70	685.70	750.48
755.85	749.85	768.87	701.23	753.14
780.73	750.00	771.54	717.73	755.26
780.82	751.58	771.66	725.56	761.40
780.89	751.76	775.36	727.60	772.89

780.97	755.32	775.52	748.23	773.31
781.11	834.91	822.45	767.16	774.11
781.15	835.04	822.65	769.70	776.80
781.21	861.89	859.60	773.75	779.68
781.27	1201.80	1140.33	776.60	781.39
781.42	1207.98	1140.46	783.09	781.84
1159.25	1208.33	1157.51	796.02	783.94
1159.41	1216.40	1159.03	816.24	822.29
1159.50	1216.51	1159.20	1164.77	1099.62
1181.78	1236.10	1167.11	1183.46	1164.58
1181.99	1236.19	1167.52	1210.77	1166.96
1182.18	1248.89	1201.71	1213.59	1171.85
1182.36	1251.69	1212.42	1213.97	1175.44
1182.57	1251.76	1212.51	1221.75	1185.52
1232.48	1329.59	1279.99	1223.47	1185.78
1232.51			1227.24	1189.27
1232.73			1240.64	1189.35
1300.14			1245.96	1222.30
			1260.70	1235.87
			1287.67	1238.35
			1345.33	1293.54

$[B_{12}F_{11}CO]^{-}$	$[B_{12}F_{11}CO]^{2-}$	$[B_{12}F_{11}NH3]^{-}$
66.04	25.09	21.35
66.07	95.71	88.87
174.26	160.00	113.88
174.27	176.75	167.40
175.57	176.86	171.59
175.62	176.91	171.95
175.84	177.41	173.41
175.86	178.97	173.63
177.31	179.52	176.82
177.40	179.74	177.15
177.44	180.13	177.57

179.09	180.18	178.76
179.14	181.36	180.61
182.03	182.41	181.48
186.46	184.86	181.61
186.53	187.04	186.42
186.96	187.19	186.49
186.99	189.94	188.70
225.98	190.31	190.47
226.72	224.87	226.35
226.78	230.17	230.35
336.34	232.59	230.83
375.88	303.59	385.49
375.96	384.30	388.34
386.71	385.77	388.89
386.87	389.07	391.26
414.83	391.75	391.31
440.27	404.10	425.63
440.28	430.77	475.73
451.66	468.86	475.90
470.71	471.49	477.62
470.81	471.84	537.59
537.35	529.57	538.90
561.36	544.23	539.28
561.45	556.07	624.40
604.77	621.88	629.61
604.85	626.48	629.63
632.73	628.44	632.47
632.84	636.71	632.97
638.91	643.58	708.72
700.64	697.09	709.77
700.73	715.74	731.84
708.44	727.78	744.85
730.89	756.68	744.96
730.99	757.10	750.79
744.75	762.89	751.53
744.79	763.14	771.40
760.43	769.30	771.91
760.63	770.25	772.65
767.49	773.30	773.04
767.61	777.21	774.45

770.18	780.14	781.59
770.30	781.31	782.95
772.49	784.45	800.64
795.27	787.23	802.09
795.38	791.74	1000.66
1152.80	1048.32	1001.81
1200.70	1159.72	1123.86
1200.82	1161.46	1173.99
1204.26	1169.21	1174.72
1209.21	1177.56	1194.89
1209.29	1180.94	1197.16
1216.15	1182.68	1198.42
1216.40	1184.15	1207.14
1238.92	1215.71	1207.28
1261.81	1227.87	1231.75
1261.92	1235.44	1251.70
1322.93	1289.72	1252.28
2192.72	1855.76	1312.06
		1399.99
		1652.44
		1652.88
		3417.48
		3518.50
		3524.02

**$[B_{12}(CN)_{12}]^{2-}$**	**$[B_{12}(CN)_{11}]^{-}$**	**$[B_{12}(CN)_{11}]^{2-}$**	**$[B_{12}(CN)_{11}H_2]^{-}$**	**$[B_{12}(CN)_{11}O_2]^{-}$ (triplet)**	**$[B_{12}(CN)_{11}O_2]^{2-}$**
82.12	78.41	81.22	56.00	54.2286	50.79
82.13	78.44	81.23	77.38	76.7398	81.40
82.14	80.64	81.94	77.46	76.9477	82.05
82.17	80.65	82.86	81.44	79.0623	82.58
82.17	82.26	82.88	81.74	80.1552	83.11
84.50	83.03	83.88	81.78	80.2547	83.59
84.51	83.70	83.93	83.31	80.463	84.34
84.54	83.72	85.61	85.02	80.624	85.63
84.55	84.52	85.65	85.23	81.5267	85.93
87.82	84.56	86.84	85.26	83.1257	87.33
87.83	85.76	88.00	86.58	83.3596	87.46
87.83	85.78	88.04	87.98	83.4947	88.18
87.85	86.25	88.80	88.66	84.1719	88.56
87.86	86.28	88.81	88.75	84.2653	88.70

89.16	87.48	89.32	90.32	84.2837	89.09
89.17	87.49	89.33	90.63	85.8672	89.36
89.18	104.88	105.96	98.91	91.7809	95.02
89.19	104.90	105.97	105.73	98.8579	100.21
108.35	106.76	109.94	105.99	101.11	103.97
108.36	198.31	202.45	106.30	102.6193	109.01
108.38	198.53	202.82	193.39	116.4703	118.83
205.29	202.27	206.68	193.50	167.3128	154.03
205.51	307.02	325.45	202.94	196.8669	205.91
205.56	307.04	325.64	318.49	204.2712	211.08
326.89	321.11	328.31	319.41	245.3669	246.68
327.03	321.19	328.40	323.81	287.9762	294.64
327.13	337.13	340.16	324.54	323.4763	325.89
327.14	345.57	356.44	337.12	323.6697	326.49
327.25	345.70	363.91	350.90	324.7896	327.20
352.50	350.17	364.02	355.17	325.3776	327.56
369.80	355.06	368.99	359.81	341.4891	344.48
369.95	355.91	372.75	360.83	352.5151	358.50
370.01	355.97	372.79	361.74	355.0572	367.74
370.17	368.67	375.14	364.48	357.4303	370.28
370.25	371.03	375.19	368.35	362.5062	370.30
370.25	371.07	379.74	369.07	365.7875	370.50
370.30	373.56	385.09	373.06	366.4611	372.49
370.35	373.62	385.12	374.12	369.1801	374.17
380.16	388.46	400.75	374.85	370.5901	376.05
380.19	388.50	400.79	391.43	375.3308	377.79
380.23	389.95	405.67	392.16	376.3135	387.53
399.30	390.05	405.69	393.69	376.9503	388.01
399.32	399.10	416.13	394.02	389.1783	399.44
399.35	399.14	416.16	404.48	391.4342	399.51
399.37	401.18	417.13	404.80	393.6852	405.51
414.94	404.69	417.15	405.94	395.388	406.19
414.96	404.71	418.45	406.62	404.3916	414.99
414.98	405.69	418.49	407.60	405.5437	415.31
415.00	405.72	419.01	408.43	405.8052	416.11
417.95	535.47	545.78	408.62	405.8784	416.80
417.98	562.35	564.28	473.53	407.7915	416.94
417.98	562.44	564.35	496.82	408.1194	418.33
418.00	585.74	603.33	540.92	409.7042	418.43

418.02	620.75	636.20	592.73	435.3031	455.61
603.10	620.82	636.24	599.59	583.7161	591.41
603.21	625.74	646.83	610.34	592.548	603.08
603.23	626.07	646.98	637.25	593.5536	607.20
652.32	628.70	653.31	639.30	641.0399	643.85
652.33	630.18	656.46	640.87	641.399	652.31
652.37	630.22	656.61	644.01	644.0913	654.39
659.59	685.11	704.94	645.23	645.8961	657.79
659.68	685.37	753.23	651.66	648.2755	658.57
659.77	694.14	753.32	680.07	651.537	662.09
659.90	711.48	759.88	698.71	653.4642	662.87
659.94	711.64	760.04	719.56	657.0615	681.35
761.45	731.31	772.10	726.15	729.9802	756.26
761.52	731.45	772.12	746.43	731.3742	759.65
761.58	746.47	773.69	748.75	746.8163	762.82
761.71	746.67	773.77	753.44	753.9106	763.23
761.74	755.08	803.15	754.04	754.3944	771.49
775.42	755.37	803.38	757.48	761.2836	772.48
775.55	831.57	811.12	765.01	764.0075	776.56
775.56	836.22	811.25	766.81	764.0675	778.88
775.66	836.44	829.67	767.35	770.6674	787.13
806.37	1081.82	1085.49	807.48	779.8026	803.09
806.55	1089.56	1085.84	816.97	787.4186	803.54
806.71	1089.73	1094.73	869.17	808.1163	807.15
806.81	1097.72	1098.86	1085.31	832.2993	823.24
1093.90	1097.79	1099.12	1088.24	904.9606	1094.79
1094.25	1110.32	1100.76	1088.75	1096.6626	1095.52
1094.39	1116.60	1101.04	1093.36	1102.1273	1095.98
1107.91	1122.25	1114.19	1099.79	1103.8991	1106.30
1107.95	1122.45	1124.16	1101.73	1106.5724	1108.04
1108.17	1138.99	1124.34	1103.73	1108.558	1109.96
1108.44	1139.00	1128.58	1106.17	1115.9814	1111.23
1108.84	2339.72	2336.27	1117.78	1118.4144	1112.70
1128.46	2340.36	2336.66	1121.32	1122.4548	1128.94
1134.94	2340.41	2336.66	1134.07	1135.3696	1133.39
1135.06	2340.45	2336.75	1296.94	1137.7081	1136.04
1135.16	2340.48	2336.76	1843.78	1140.3707	1139.86
2340.59	2343.38	2338.50	2338.63	1334.3225	1192.17
2341.42	2343.69	2338.71	2339.27	2281.8361	2339.76

2341.43	2343.80	2338.73	2339.40	2340.3648	2340.24
2341.44	2343.83	2338.74	2339.56	2341.1861	2340.34
2341.45	2344.06	2338.75	2339.58	2342.7015	2340.46
2341.45	2344.06	2340.40	2341.46	2342.8858	2340.61
2341.59			2341.77	2343.2431	2340.91
2341.61			2341.78	2346.2894	2340.98
2341.61			2341.99	2346.602	2341.00
2341.73			2342.00	2346.6582	2341.03
2341.75			2342.35	2346.8214	2341.32
2341.75			3336.68	2347.0098	2341.40

$[B_{12}(CN)_{11}CO]^{-}$	$[B_{12}(CN)_{11}CO]^{2-}$	$[B_{12}(CN)_{11}NH_3]^{-}$	$[B_{12}(CN)_{11}NH_3]^{2-}$
66.30	18.76	25.23	56.48
66.35	81.46	67.43	59.57
75.66	82.00	72.27	72.17
75.69	82.16	74.99	75.82
79.82	82.47	78.66	76.23
79.86	83.23	79.66	79.40
79.87	84.34	80.21	80.75
80.70	84.89	80.35	81.48
80.72	84.92	81.37	81.96
80.84	85.79	81.54	83.68
82.91	87.67	82.23	86.65
82.95	87.84	83.91	87.17
83.76	87.92	83.96	87.47
83.78	88.39	84.06	87.88
84.34	88.80	84.17	92.87
84.35	89.28	93.00	96.70
86.89	89.41	99.09	102.09
86.90	91.51	99.28	103.06
100.35	104.20	102.28	106.60
103.06	107.40	104.41	115.06
103.08	109.35	134.96	132.97
197.10	150.42	141.28	141.11

197.29	202.22	199.00	208.90
197.63	206.24	246.75	247.77
315.19	230.30	249.63	250.03
325.07	295.92	324.29	278.60
325.17	326.25	324.38	297.10
325.50	326.35	326.59	301.94
325.65	327.08	326.59	312.97
350.90	328.44	339.61	323.81
357.46	344.96	350.26	326.30
357.49	358.56	357.81	333.71
362.18	368.44	357.91	334.84
363.40	369.18	364.09	345.53
364.59	369.82	371.36	353.84
364.60	369.84	371.82	354.21
369.48	370.67	374.40	370.61
369.58	374.93	374.54	376.41
374.20	375.71	377.72	376.54
374.23	378.56	377.84	377.28
377.58	386.01	383.52	377.83
391.18	386.04	400.08	382.47
391.23	398.45	400.13	393.57
393.45	399.68	400.83	403.10
393.48	405.27	401.07	407.30
405.05	405.45	408.48	408.08
405.07	415.16	408.56	417.14
405.88	415.29	412.81	417.64
405.90	416.66	413.09	419.50
408.65	416.88	416.30	422.44
408.66	417.69	417.32	424.27
409.58	418.19	421.13	426.05
454.82	418.53	432.03	430.33
454.87	447.79	586.64	566.76
593.83	581.89	587.35	583.09
599.50	601.40	597.23	585.49
599.61	603.57	638.43	596.37
645.43	638.00	638.57	614.35
645.46	647.29	653.33	618.29
646.57	654.60	653.68	620.93
646.75	655.20	655.85	625.83

649.19	655.68	660.97	637.39
656.20	659.14	661.02	639.95
664.94	659.40	668.11	640.45
665.10	664.21	753.57	647.86
736.11	754.83	753.65	665.83
736.17	757.84	762.43	670.46
758.88	759.35	762.62	717.72
758.99	760.71	768.34	721.94
760.10	764.22	768.51	731.12
764.10	767.42	773.75	742.77
764.20	771.83	776.67	748.58
773.44	774.91	776.93	753.45
773.51	776.68	804.43	769.30
790.21	803.49	804.53	773.05
790.27	803.79	813.15	789.86
820.58	808.23	814.50	804.61
820.74	810.11	1011.32	993.21
1069.93	1030.95	1011.70	994.22
1098.70	1090.04	1089.00	1052.28
1100.97	1091.90	1102.49	1064.14
1101.40	1097.21	1102.90	1072.04
1107.61	1101.30	1110.11	1074.71
1107.91	1103.88	1113.20	1077.04
1113.85	1106.50	1113.99	1084.82
1114.28	1107.25	1121.61	1116.80
1120.58	1120.24	1121.97	1125.98
1137.15	1128.60	1135.44	1133.20
1139.44	1130.33	1140.02	1139.14
1139.64	1134.97	1152.81	1144.08
2292.27	1919.19	1153.34	1146.88
2345.03	2338.92	1471.98	1441.82
2345.70	2339.28	1647.05	1630.30
2345.71	2339.61	1647.70	1645.73
2345.77	2339.96	2333.10	2252.71
2345.79	2340.15	2334.55	2265.01
2347.72	2340.25	2335.68	2266.95
2348.03	2340.48	2337.13	2272.33
2348.11	2340.54	2337.59	2287.85
2348.11	2340.66	2346.69	2299.39

2348.39	2340.75	2347.19	2303.04
2348.40	2340.81	2347.24	2304.16
		2347.4	2305.5
		2347.4	2313.8
		2348.6	2325.8
		3381.6	3377.7
		3470.1	3457.1
		3478.9	3487.0
